# PRO40 Is a Scaffold Protein of the Cell Wall Integrity Pathway, Linking the MAP Kinase Module to the Upstream Activator Protein Kinase C

**DOI:** 10.1371/journal.pgen.1004582

**Published:** 2014-09-04

**Authors:** Ines Teichert, Eva Katharina Steffens, Nicole Schnaß, Benjamin Fränzel, Christoph Krisp, Dirk A. Wolters, Ulrich Kück

**Affiliations:** 1 Department for General and Molecular Botany, Ruhr-University Bochum, Bochum, Germany; 2 Department of Analytical Chemistry, Ruhr-University Bochum, Bochum, Germany; Duke University Medical Center, United States of America

## Abstract

Mitogen-activated protein kinase (MAPK) pathways are crucial signaling instruments in eukaryotes. Most ascomycetes possess three MAPK modules that are involved in key developmental processes like sexual propagation or pathogenesis. However, the regulation of these modules by adapters or scaffolds is largely unknown. Here, we studied the function of the cell wall integrity (CWI) MAPK module in the model fungus *Sordaria macrospora*. Using a forward genetic approach, we found that sterile mutant pro30 has a mutated *mik1* gene that encodes the MAPK kinase kinase (MAPKKK) of the proposed CWI pathway. We generated single deletion mutants lacking MAPKKK MIK1, MAPK kinase (MAPKK) MEK1, or MAPK MAK1 and found them all to be sterile, cell fusion-deficient and highly impaired in vegetative growth and cell wall stress response. By searching for MEK1 interaction partners via tandem affinity purification and mass spectrometry, we identified previously characterized developmental protein PRO40 as a MEK1 interaction partner. Although fungal PRO40 homologs have been implicated in diverse developmental processes, their molecular function is currently unknown. Extensive affinity purification, mass spectrometry, and yeast two-hybrid experiments showed that PRO40 is able to bind MIK1, MEK1, and the upstream activator protein kinase C (PKC1). We further found that the PRO40 N-terminal disordered region and the central region encompassing a WW interaction domain are sufficient to govern interaction with MEK1. Most importantly, time- and stress-dependent phosphorylation studies showed that PRO40 is required for MAK1 activity. The sum of our results implies that PRO40 is a scaffold protein for the CWI pathway, linking the MAPK module to the upstream activator PKC1. Our data provide important insights into the mechanistic role of a protein that has been implicated in sexual and asexual development, cell fusion, symbiosis, and pathogenicity in different fungal systems.

## Author Summary

The specific response to environmental cues is crucial for cell differentiation and is often mediated by highly conserved eukaryotic MAP kinase (MAPK) pathways. How these pathways react specifically to huge numbers of different cues is still unclear, and current literature about adapter and scaffolding proteins remains scarce. However, gaining fundamental insight into molecular signaling determinants is pivotal for combating diseases with impaired signal transduction processes, such as Alzheimer's disease or cancer. Importantly, signal transduction can easily be studied in lower eukaryotes like filamentous fungi that are readily genetically tractable. The fungus *Sordaria macrospora* has a long history as an ideal model system for cell differentiation, and we show here that the proposed cell wall integrity (CWI) MAPK module of this fungus controls differentiation of sexual fruiting bodies, cell fusion, polar growth and cell wall stress response. We further discovered that developmental protein PRO40 binds the MAPK kinase kinase (MAPKKK), the MAPK kinase (MAPKK) and upstream activator protein kinase C (PKC1) of the CWI pathway and is required for MAK1 activity, thereby providing evidence that PRO40 is a scaffold protein. Collectively, our findings reveal a molecular role for a protein implicated in development, cell fusion, symbiosis, and pathogenicity in different fungi.

## Introduction

Mitogen-activated protein kinase (MAPK) cascades are central components of signaling networks in all eukaryotic organisms [1]–[3]. They consist of a three-tiered module containing a MAPK kinase kinase (MAPKKK), a MAPK kinase (MAPKK), and a MAPK, each activating the subsequent one via phosphorylation. MAPK signaling has been extensively studied in the yeast *Saccharomyces cerevisiae*, in which five MAPKs have been reported (reviewed in [4]). Three MAPK modules have also been identified in most filamentous fungi, including *Aspergillus fumigatus*, *Magnaporthe grisea*, *Neurospora crassa*, and *Sordaria macrospora* [5], [6]. Based on homology to *S. cerevisiae*, they supposedly constitute a cell wall integrity (CWI), pheromone signaling (PS), and osmosensing cascade. Notably, CWI pathway components have been studied in various fungi and are known to be not only responsible for cell wall stress response. For example, in *A. fumigatus*, the CWI pathway is involved in pyomelanin and gliotoxin formation, response to reactive oxygen species and siderophore biosynthesis [7], [8]. The CWI pathway of *N. crassa* is necessary for polar growth, conidiation, fusion of conidial germling protrusions (CATs; conidial anastomosis tubes), and fruiting body formation [9]–[11]. CWI MAPKs from *Cochliobolus heterostrophus*, *Coniothyrium minitans*, *Fusarium graminearum*, and *Magnaporthe oryzae* have further been shown to be involved in female fertility, heterokaryon formation, mycoparasitism and pathogenicity [12]–[15].

Scaffold and adapter proteins are vital for the spatiotemporally correct assembly and signaling output of MAPK pathways and are involved in decision making, thereby enabling highly specific adaptation of signaling pathways [16]–[19]. However, despite many studies already addressing the role of the CWI pathway in fungal development, little is still known about the coordination of this pathway as well as the regulation of specific responses. Further, in *S. cerevisiae*, the polarisome component Spa2p is known to act as a scaffold-like protein for the CWI MAPKK and MAPK during polar growth [20]. However, information about scaffold or adapter proteins of the CWI pathway is still lacking for filamentous fungi.

In this study, we analyzed the CWI pathway of the model fungus *S. macrospora*. This filamentous ascomycete has four major advantages over other fungal systems for the study of sexual development (reviewed in [21], [22]). First, it rapidly forms mature fruiting bodies (perithecia) within 7 days. Second, it is self-fertile (homothallic) and thus does not need a mating partner. Moreover, the sexual phenotypes are immediately recognizable. Third, it does not form aerial hyphae with vegetative spores (conidia), which overgrow small pre-fruiting structures like the ascogonial coils (10–20 µm) or the spherical protoperithecia (20–90 µm) and thus prevent their observation. Forth, a collection of developmental *S. macrospora* mutants is available and well characterized showing defects at different stages of sexual development. Recent analyses have specifically focused on ‘pro’ mutants generating only protoperithecia, a stage the wildtype reaches after 3–4 days. Characterizing these ‘pro’ mutants has led to the identification of several developmental proteins [23]–[28].

Using a forward genetic approach, we identified mutant pro30 as a CWI pathway mutant and generated three CWI kinase deletion strains for functional studies regarding sexual development, cell fusion, vegetative growth, and cell wall stress response. Affinity purification of CWI MAPKK MEK1 revealed that this kinase interacts with the developmental protein PRO40, a protein essential for sexual development and cell fusion [24]. Using further affinity purification-mass spectrometry and yeast two-hybrid analyses, we show that PRO40 binds to MAPKKK MIK1, MAPKK MEK1, and the upstream activator protein kinase C (PKC1). Phosphorylation studies revealed that PRO40 is required for correct signaling via the CWI pathway. Here, we propose a new model in which PRO40 acts as a scaffold protein for the CWI pathway, linking the MAPK module to its upstream activators.

## Results

### Sterile pro30 carries a mutated *mik1* gene encoding the CWI MAPKKK

**Figure 1:**
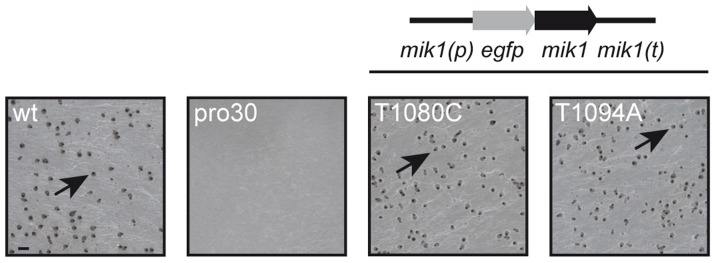
Complementation of pro30 with full-length *mik1*. Strains were grown on BMM plates for 7 days. Mutant pro30 was complemented with *gfp-mik1* controlled by the *mik1* promoter (T1080C, T1094A). Mature perithecia (arrows) can be observed in the wildtype and complemented strains. Scale bar, 1 mm.

To identify regulators of fruiting body formation, we previously generated a large collection of developmental *S. macrospora* mutants [22], [25]. One class of mutants was named with the prefix ‘pro’, because these mutants develop only protoperithecia. In this study, we analyzed the underlying mutation in mutant pro30 by next-generation sequencing as described recently [25] (Table S1, SRX483430). SNP analysis revealed a C to T transition at position 904 of the *SMAC_03673* gene in the pro30 mutant genome, resulting in a Q302stop substitution at the protein level (Figure S1). Progeny from a cross of pro30 to fus were analyzed, and the mutation was found to strictly co-segregate with the sterile phenotype (Figure S1). *SMAC_03673* encodes a 1714 amino acid protein that exhibits 88.4% identity to *N. crassa* CWI MAPKKK MIK1 (NCU02234, XP_959647.2) and 21.3% identity to *S. cerevisiae* CWI MAPKKK Bck1p (EWG95039.1, Figure S9), as revealed by BLAST searches [29]. *SMAC_03673* was therefore renamed *mik1*. To confirm that the C to T transition in *mik1* is responsible for the mutant phenotype, we transformed pro30 with a full-length copy of *mik1*. As can be clearly seen from Figure 1, pro30 transformants regained the ability to form perithecia. Thus, a functional MIK1 MAPKKK is required for sexual development in *S. macrospora*.

### MIK1, MEK1, and MAK1 are involved in sexual development and hyphal fusion

The finding that the MIK1 MAPKKK is required for sexual development in pro30 prompted us to analyze the role of CWI pathway kinases in sexual development in more detail. BLAST searches [29] against the *N. crassa* genome sequence (http://www.broadinstitute.org/annotation/genome/neurospora/MultiHome.html) [30] and the *S. cerevisiae* genome sequence (http://www.yeastgenome.org/) [31] revealed that MAPKK SMAC_02183 (CCC11961.1) and MAPK SMAC_05504 (CCC12327.1) are homologous to *N. crassa* MEK-1/*S. cerevisiae* Mkk1p and Mkk2p (Figure S10) and *N. crassa* MAK-1/*S. cerevisiae* Mpk1p (Figure S11), respectively. We subsequently renamed *SMAC_02183* and *SMAC_05504 mek1* and *mak1*, respectively. Deletion strains for *mik1*, *mek1*, and *mak1* were generated by homologous recombination as described previously using *S. macrospora* Δku70 as host [32]. For generating deletion strains devoid of the Δku70 background, PCR-verified primary transformants were crossed to spore color mutant fus or to sterile mutant pro40 [24], [25]. Subsequent single spore isolation led to Δmik1, Δmek1 and Δmak1 single deletions as verified by PCR and Southern blot analysis (Figure S2, Figure S3, and Figure S4).

**Figure 2:**
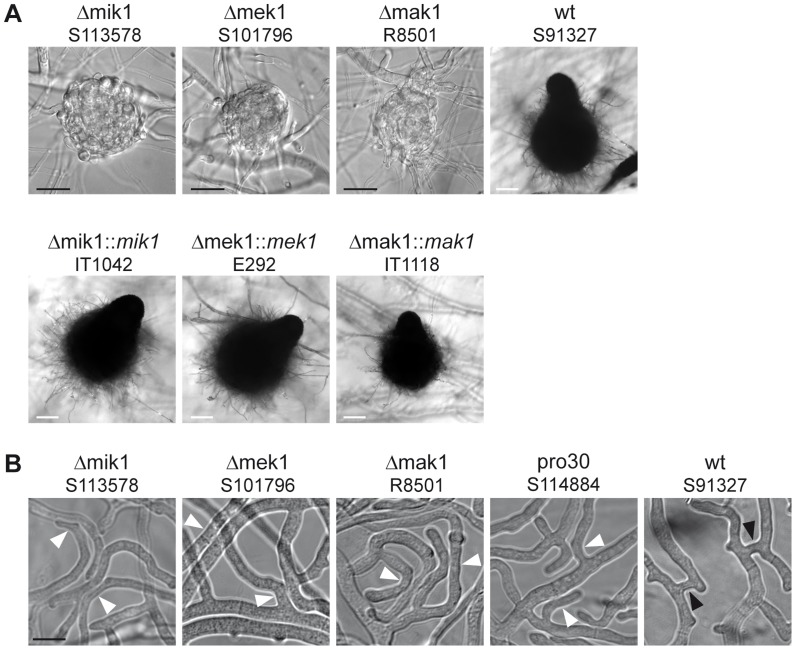
Phenotypic characterization of kinase deletion strains. (A) Sexual development was assayed after 7 days of growth on BMM slides. Mature perithecia are only generated in wildtype (wt) and complemented deletion strains (Δmik1::*mik1*, Δmek1::*mek1*, and Δmak1::*mak1*). Δmik1, Δmek1, and Δmak1 generate only protoperithecia. White scale bar, 100 µm; black scale bar, 20 µm. (B) DIC microscopy of hyphal fusion in subperipheral regions. Strains were grown on solid MMS with a cellophane layer for 2 days. Hyphal fusion bridges are indicated by black arrowheads, whereas hyphae that grow in close contact without fusion are indicated by white arrowheads. Scale bar, 10 µm.

We compared sexual development of the three different kinase deletion strains to wildtype. Figure 2A shows sexual structures generated after 7 days of growth on BMM. Like pro30, the kinase deletion mutants did not develop further after protoperithecium formation. Note that mature perithecia were never observed, even after prolonged incubation (Figure 2A). However, sexual development in the deletion strains was re-established by introducing wildtype copies of the deleted genes. Recently, the sexual structures of *N. crassa* Δmik1, Δmek1, and Δmak1 mutants were described as difficult to detect due to early-onset autolysis [9]. Although we observed autolysis in *S. macrospora* Δmik1, Δmek1, and Δmak1, protoperithecia were found frequently in Δmik1 and Δmek1 (>200 protoperithecia per microscope slide). It should be noted, however, that we rarely found protoperithecia in Δmak1 (2 protoperithecia on average per microscope slide; data not shown).

A general observation made by ourselves and others is that a defect in sexual development is often linked to a defect in hyphal fusion [23], [33]–[36]. We therefore examined pro30, Δmik1, Δmek1, and Δmak1 for the occurrence of fusion events between vegetative hyphae. Fusion bridges were frequently observed in the wildtype by light microscopy (Figure 2B). However, we were unable to detect fusion bridges in the three kinase deletion mutants as well as in pro30, although hyphae frequently made contact (Figure 2B).

### MIK1, MEK1, and MAK1 constitute the *S. macrospora* CWI module

**Figure 3:**
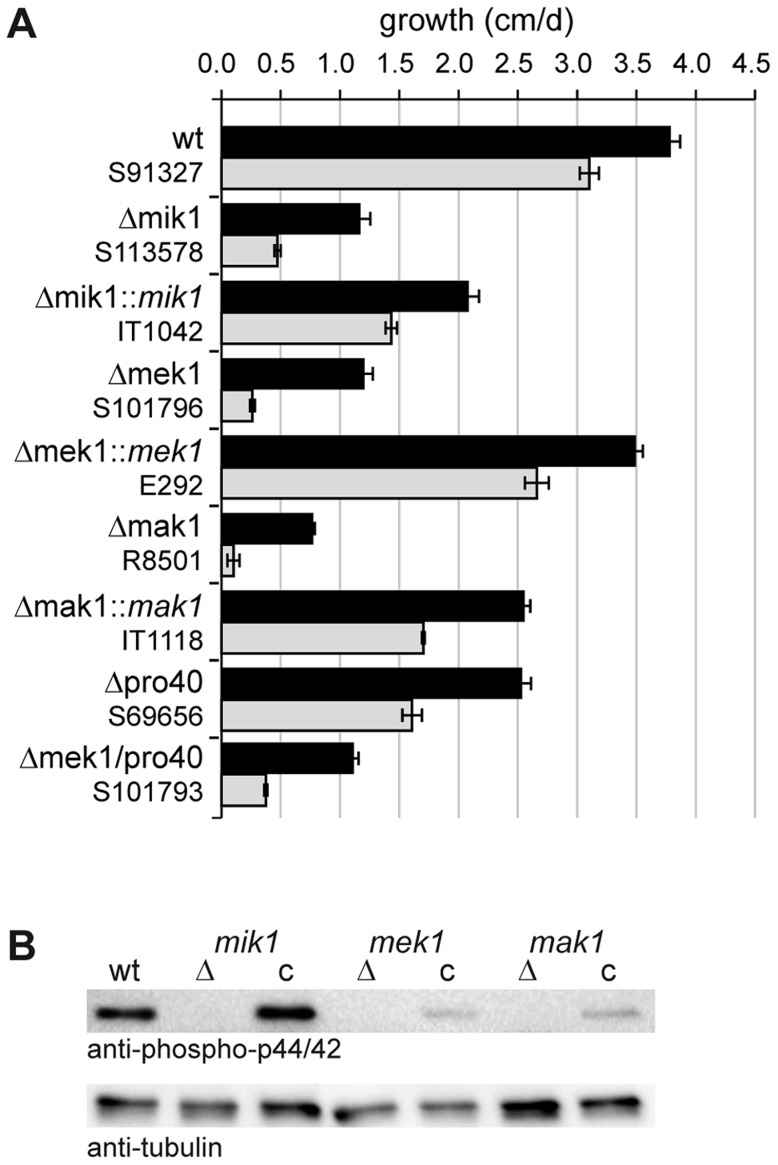
Growth tests and phosphorylation studies. (A) Vegetative growth and cell wall stress response of CWI kinase mutants. Sensitivity to Calcofluor White (CFW, 250 µg/ml) was monitored in race tubes for 7 consecutive days. Average growth rates on SWG (black bars) and SWG + CFW (gray bars) and standard deviations from three independent experiments are shown. (B) MAK1 phosphorylation was studied in wildtype (wt), deletion (Δ) and complemented (c) strains by Western blot analysis with anti-phospho-p42/44 antibody. Complemented strains are Δmik1::*mik1*, IT1042; Δmek1::*mek1*, E292; Δmak1::*mak1*, IT1118. A representative example of three independent experiments is shown. MAK1 is not phosphorylated in any of the deletion strains. Phosphorylation is reconstituted after reintroduction of full-length gene copies of *mik1*, *mek1*, and *mak1* into the respective deletion strains. The signal for tubulin was used as internal standard.

To evaluate the role of MIK1, MEK1, and MAK1 in cell wall stress response, we performed growth tests on medium containing Calcofluor White (CFW). CFW is a common agent used to test fungal mutants for cell wall stress-related defects [37]. We assessed growth during 7 consecutive days in race tubes on synthetic SWG medium ± CFW. Vegetative growth of Δmik1, Δmek1, and Δmak1 was severely impaired even without CFW and was reduced by 70–80% in comparison to wildtype. Figure 3A shows mean values of average growth rates from three independent experiments. Growth of the wildtype on SWG + CFW (gray bar) was reduced by 19% compared to growth on SWG. A much more drastic effect of CFW on vegetative growth was observed in the kinase deletion strains. Growth in these strains was reduced by 62–91% in the presence of CFW (Figure 3A, gray bars), compared to growth on SWG (Figure 3A, black bars). Integration of wildtype copies of *mik1*, *mek1*, and *mak1* into the respective deletion strains partially complemented the growth defect. This result may be explained by mis-expression of the kinase genes from the constitutive *A. nidulans gpd* promoter (*mik1* and *mek1*) and the inducible *S. macrospora Smxyl* promoter (*mak1*) [38], [39].

**Figure 4:**
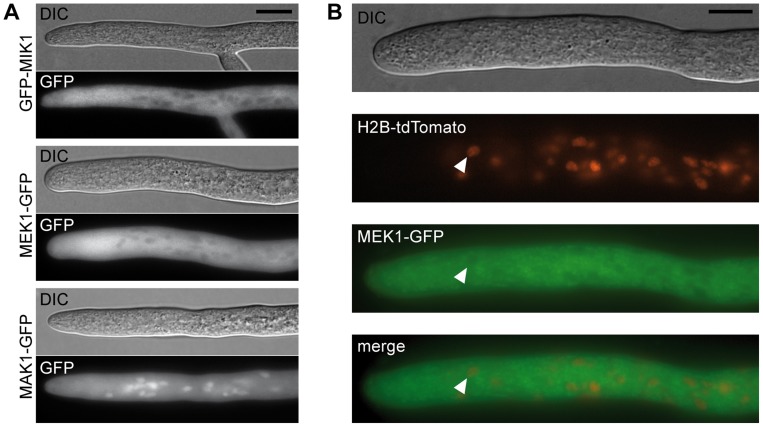
Localization of MIK1, MEK1, and MAK1. (A) GFP-labeled MIK1 and MEK1 are present in the cytoplasm and absent from nuclei. GFP-labeled MAK1 localizes to the cytoplasm, but is also targeted to the nucleus. (B) Co-localization experiments with MEK1-GFP and H2B-tdTomato show that spherical organelles devoid of MEK1 labeling are nuclei (arrowheads). Scale bar, 10 µm.

Signal transduction via a MAPK module requires several subsequent phosphorylation events, eventually leading to phosphorylation of the MAPK. Western blot analysis with deletion mutants and complemented strains showed that MIK1 and MEK1 are required for MAK1 phosphorylation, confirming the composition of the three-tiered MAPK module (Figure 3B). We further analyzed the localization of MIK1, MEK1, and MAK1 by generating GFP fusions. Functionality of the fusion proteins was confirmed by complementation of the corresponding deletion strains. Fluorescence microscopy showed that MIK1 and MEK1 reside in the cytoplasm and are clearly absent from spherical organelles (Figure 4A). Co-localization experiments using strains with MEK1-GFP and tdTomato-tagged histone H2B identified these organelles to be nuclei (Figure 4B). MAK1-GFP localized to the cytoplasm and the nucleus. This localization pattern is consistent with previously published data, e.g. from the fission yeast *Schizosaccharomyces pombe*, where MAPKKK Mkh1 and MAPKK Pek1 localize to the cytoplasm, whereas MAPK Pmk1 shuttles between the cytoplasm and the nucleus [40].

### Affinity purification and mass spectrometry reveals developmental protein PRO40 as the MEK1 interaction partner

As mentioned above, little is known about the regulation of the fungal CWI pathway through scaffolds or adapters. We surmised that these regulators might be identified by searching for interaction partners of the kinases. In a recent transcriptomics analysis, we found the *mek1* transcript among the most abundant transcripts in protoperithecia [41], and therefore were prompted to search for MEK1 interaction partners.

MEK1 was fused to an N-terminal tandem affinity purification (TAP) tag consisting of protein A, a TEV protease cleavage site and the calmodulin-binding peptide [42], [43]. TAP in combination with mass spectrometry using multi-dimensional protein identification technology (MudPIT) [44], [45] facilitates the detection of low-abundant proteins. This approach has been applied successfully to the identification of *S. macrospora* proteins from complex mixtures, and yields a huge number of peptides even after tandem purification [43].

Functionality of NTAP-MEK1 was shown by complementation analysis (Figure 2A), and single spore isolate E292 that expressed high levels of the fusion protein (Figure S5) was chosen for TAP-MudPIT. Proteins that were identified with at least two different peptides in at least two out of four replicate experiments are listed in Table S2. Notably, the most abundant protein detected by MudPIT was MEK1 itself. We further detected other members of the CWI pathway, namely MIK1 and upstream components protein kinase C (PKC1, SMAC_04666; CCC11683.1) and small GTPase RHO1 (SMAC_06239; CCC07244.1). Strikingly, one of the most abundant proteins detected in the MEK1 TAP-MudPIT analysis was PRO40 (CCC06426.1; Table S2). This protein has previously been shown to be involved in sexual development and hyphal fusion [24]. Fungal PRO40 homologs have been connected to sexual and asexual development, cell fusion, symbiosis, and pathogenicity, but their exact molecular function is still currently unknown [35], [46]–[48].

To verify the MEK1-PRO40 interaction, we performed affinity purification of FLAG-tagged PRO40 (FLAG-AP) followed by MudPIT. The PRO40-FLAG fusion construct has already been shown to complement pro40 [24]. For FLAG-AP, we used strain T184.2NS11 (Δpro40::*pro40-3xFLAG*) yielding detectable amounts of PRO40-FLAG in the eluate (Figure S5).

FLAG-AP in combination with MudPIT was performed three times with T184.2NS11 and twice with wildtype (control). A full list of proteins detected with at least two different peptides in at least two out of three (PRO40-FLAG) or two out of two (wildtype) replicate experiments is given in Tables S3 and S4, respectively. Due to the single FLAG purification step, the number of identified proteins in the PRO40-FLAG dataset was even larger (444 proteins) than the number of proteins identified in the TAP-MEK1 dataset (308 proteins). However, five proteins were identified consistently in all three experiments with PRO40-FLAG, but not with wildtype control experiments. Of these five proteins, three have been assigned functions in cell differentiation. PKC1 is an upstream activator of the CWI module, COP9-2 (SMAC_01284; CCC07717.1) is a subunit of the COP9 signalosome, which has been described to regulate sexual development in *A. nidulans*, and PRO4/LEU1 (SMAC_07082; CCC13458.1) is an enzyme that is involved in the leucine biosynthesis pathway, which is essential for fruiting body formation in *S. macrospora* [49]–[51]. Since we detected PRO40 in one of the wildtype control experiments (Table S4), we calculated ratios between the spectral counts in PRO40-FLAG and control experiments to evaluate the specificity of such proteins detected in both experiment types (see Materials and Methods, Table S5). This approach reduced the number of high-confidence hits for PRO40 interaction partners to 123 proteins, including MEK1 and RHO1.

In addition to TAP-MudPIT with NTAP-MEK1 in a Δmek1 background, we also performed TAP-MudPIT in a Δpro40 background (strain E2544; Δpro40::NTAP-MEK1; Figure S5). Proteins that were identified with at least two different peptides in at least two out of three replicate experiments were considered as high-confidence interactors (Table S2). Among these, we identified MIK1 and RHO1, but not PKC1. Interestingly, MEK1 seems to interact with the Woronin body protein HEX1 (SMAC_01601; CCC08037.1) independent of whether or not PRO40 is present, since HEX1 was detected in both the Δmek1::NTAP-MEK1 and the Δpro40::NTAP-MEK1 datasets (Table S2).

**Figure 5:**
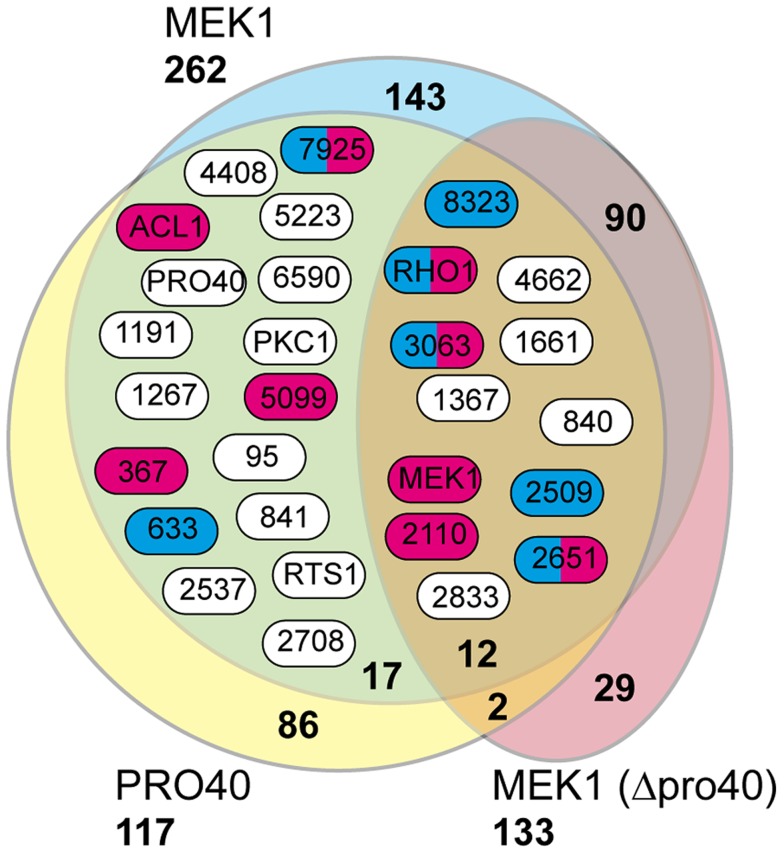
Shared interaction network of MEK1 and PRO40. Venn diagram comparing the three datasets generated from affinity purification and mass spectrometry with strains Δpro40::PRO40-FLAG (PRO40), Δmek1::NTAP-MEK1 (MEK1), and Δpro40::NTAP-MEK1 (MEK1(Δpro40)). The 12 proteins found in all three datasets and the 17 proteins found only in the PRO40 and MEK1 datasets are represented by boxes with *S. macrospora* locus tag numbers or protein designations. The magenta and blue box color indicates that the transcript of the encoding gene belongs to the top500 transcripts (with respect to read counts) in protoperithecia and vegetative hyphae, respectively (data taken from a previous transcriptomics analysis [41]).

From our results, we subtracted known background (a list of proteins considered background derived from numerous unrelated affinity purification-MS experiments is provided in Table S6), and by comparing the three datasets (Δpro40::PRO40-FLAG, Δmek1::NTAP-MEK1, and Δpro40::NTAP-MEK1) found overlaps between the PRO40 and MEK1 interaction networks (Figure 5, Table 1). Specifically, we found 12 proteins in all three datasets. From the CWI pathway, this group contains MEK1 and RHO1. Another 17 proteins appeared in the Δpro40::PRO40-FLAG and Δmek1::NTAP-MEK1 datasets, but not in the Δpro40::NTAP-MEK1 dataset (Figure 5, Table 1), indicating that interaction of MEK1 with these 17 proteins depends on the presence of PRO40. Among these 17 proteins was PKC1, ATP citrate lyase ACL1 (SMAC_06775; CCC07573.1), previously found to be involved in *S. macrospora* sexual development [52], and a putative regulatory subunit of protein phosphatase PP2A, RTS1 (SMAC_02633; CCC10054.1). This regulatory subunit is of high interest because of a recently described fungal striatin-interacting phosphatase and kinase (STRIPAK) complex, containing protein phosphatase 2A (PP2A) and several ‘PRO’ proteins [43].

**Table 1:** Shared interaction partners of MEK1 and PRO40.

*Sm*	MEK1 (Δmek1)			PRO40			MEK1 (Δpro40)			Description
SMAC_	#s	#p	cov	#s	#p	cov	#s	#p	cov	
00095	9	6	16.0	25	9	26.5	-	-	-	Eukaryotic translation initiation factor 3
00367	11	7	29.0	36	10	32.4	-	-	-	Eukaryotic translation initiation factor
00633	4	3	7.8	33	11	35.6	-	-	-	Pci domain protein
00840	98	45	36.84	36	21	19.4	18	11	10.2	Fatty acid synthase beta subunit dehydratase
00841	57	34	27.6	35	20	14.5	-	-	-	Fatty acid synthase alpha subunit
01191	7	6	42.1	37	10	53.1	-	-	-	Short chain dehydrogenase
01267	5	4	7.8	25	11	20.7	-	-	-	Hsp70 family protein
01367	8	4	9.7	94	13	29.0	8	3	6.9	Fasciclin domain family protein
01661	11	4	16.2	52	8	25.5	9	3	13.3	2-Nitropropane dioxygenase
02110	18	10	14.9	58	19	30.7	9	7	12.9	Eukaryotic translation initiation factor 3
02183	749	31	61.3	17	7	19.7	2135	29	60.0	MEK1
02509	7	3	27.9	25	3	26.5	7	2	14.7	60 s ribosomal protein l27e
02537	5	4	7.5	7	4	8.3	-	-	-	Xpg i-region protein
02633	5	5	12.6	24	9	20.3	-	-	-	PP2A regulatory subunit
02651	12	6	42.9	12	4	36.4	11	4	32.9	60 s ribosomal protein
02708	11	5	19.9	17	6	29.1	-	-	-	Eukaryotic translation initiation factor 3 subunit f
02833	6	6	13.1	4	4	8.1	5	4	7.8	Duf1750 domain containing protein
03063	13	7	11.8	34	11	16.9	10	4	8.0	Oligopeptide transporter
04408	12	6	21.4	25	11	36.9	-	-	-	Glycogen synthase kinase
04662	19	10	22.4	10	8	14.4	18	9	15.3	Topoisomerase II-associated protein pat1
04666	7	4	4.4	18	10	11.7	-	-	-	PKC1
04815	342	10	13.8	5913	68	60.3	-	-	-	PRO40
05099	9	6	14.2	29	12	31.8	-	-	-	ATP-binding cassette sub-family f member 2
05223	6	4	9.0	5	4	14.0	-	-	-	Malate synthase
06239	8	3	28.6	31	4	29.1	5	2	16.3	RHO1
06590	6	5	9.3	5	4	5.7	-	-	-	Rho-GTPase-activating protein 8
06775	28	14	34.2	53	20	45.9	-	-	-	ATP citrate lyase 1
07925	12	7	25.7	53	5	21.7	-	-	-	Polyubiquitin
08323	110	7	43.5	24	3	15.2	209	4	40.6	Histone H2B

#s, number of spectral counts; #p, number of peptide counts; cov, coverage (%). *Sm*, *S. macrospora*.

As mentioned above, we recently performed a transcriptomics analysis of protoperithecia by laser microdissection and RNA-seq [41]. When we superimposed data from this study on the Venn diagram in Figure 5, we found that the group of interaction partners shared by PRO40 and MEK1 contains proteins whose transcripts belong to the top500 genes (with respect to read counts) in either vegetative growth conditions (blue) or protoperithecia (magenta). For example, besides the *mek1* transcript, the *rho1* and *acl1* transcripts are in the top500 list in protoperithecia (Figure 5; [41]). These data highlight the significance of the MEK1-PRO40 interaction network for fungal sexual development.

### Yeast two-hybrid analysis points to the presence of CWI sub-complexes

We further searched for PRO40 interaction partners by performing yeast two-hybrid assays with full-length PRO40 as bait. For prey, we generated two *S. macrospora* Matchmaker libraries using cDNA derived from different cultures to include cDNA from vegetatively and sexually propagating mycelia. Screening of 10^7^ yeast cells yielded 1600 clones with *ade* and *his* reporter gene activity, and 96 clones additionally showing *lacZ* reporter gene activity in two subsequent assays were subjected to DNA isolation and sequence analysis, which led to the identification of 13 different genes (Table S7). 11 of the 96 clones carried *mek1* sequences. To assess the strength of the PRO40-MEK1 interaction, we performed quantitative β-galactosidase assays with a strain carrying BD-PRO40 and AD-MEK1_v01. Note that MEK1-v01 corresponds to an N-terminally truncated MEK1, which is due to an annotation error in the *S. macrospora* genome version 01 vs. version 02 [5], [41]. Mean values of β-galactosidase activity of three independent experiments were 86.7±16.9 U/mg protein compared to 5.0±2.4 U/mg protein for the control experiment (BD-PRO40 and AD), indicating a strong interaction between PRO40 and MEK1.

**Figure 6:**
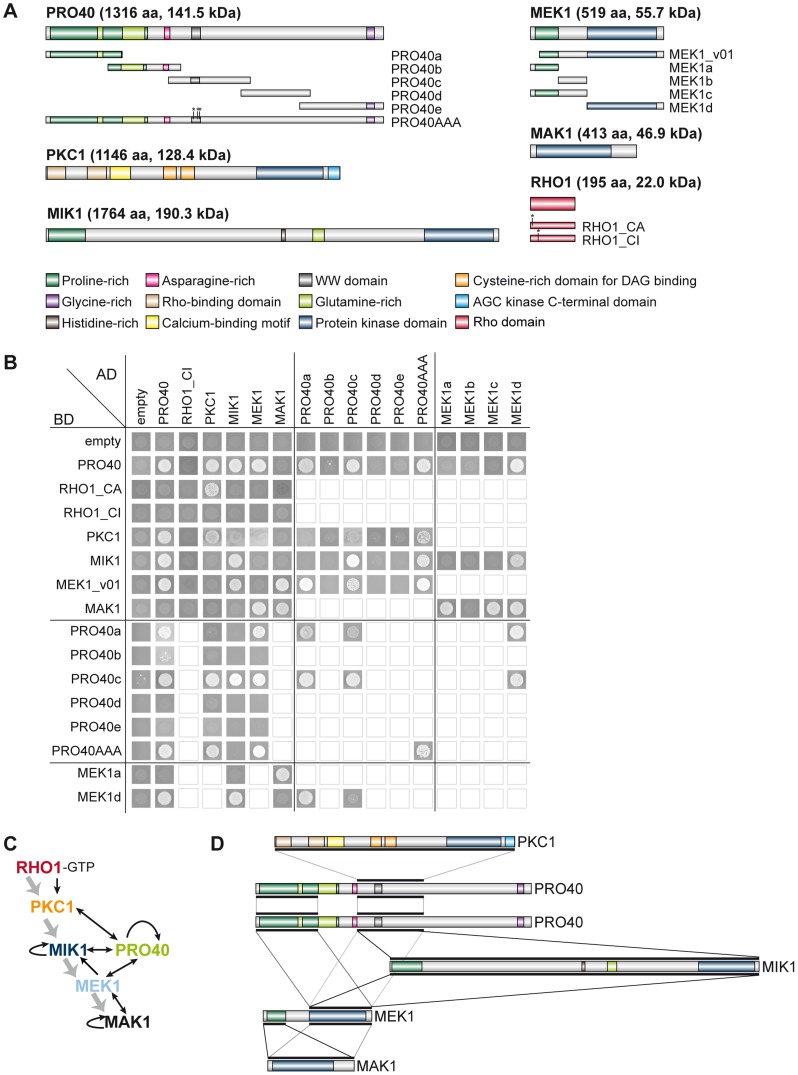
Interactions of PRO40 with components of the CWI pathway. (A) Structures of proteins used for yeast two-hybrid analysis. Derivatives generated in addition to full-length constructs are shown below the protein structures. (B) Yeast two-hybrid analysis of CWI pathway components and PRO40. Yeast cells were drop-plated on SD medium lacking leucine, tryptophan, histidine, and adenine. Empty squares indicate that interactions were not tested. (C) Schematic overview of signal transduction and protein-protein interactions within the PRO40-CWI complex. Signaling through the pathway is depicted by gray arrows; interactions are depicted by black arrows. (D) Interaction sites between PRO40, PKC1, MIK1, MEK1, and MAK1. Black bars represent interaction sites tested in yeast two-hybrid analyses (A, B). For reasons of clarity, only PRO40 is depicted as homodimer.

To gain further insight into the protein interactions within the deduced multi-subunit complex, we tested reciprocal interaction between the six proteins, PRO40, RHO1, PKC1, MIK1, MEK1, and MAK1, in a yeast two-hybrid assay. We used constitutively active (RHO1_CA) and inactive (RHO1_CI) versions of RHO1 to determine interactions dependent on RHO1 activity. Protein structures of all tested proteins are given in Figure 6A. Mating of yeast strains carrying GAL4-AD and -BD translational fusions with the abovementioned proteins resulted in diploid cells that were tested for reporter gene activity. As can be seen from growth of yeast colonies on SD medium lacking adenine and histidine, PRO40 interacted with PKC1, MIK1, and MEK1, but not RHO1 and MAK1 (Figure 6B; A growth control of yeast colonies is shown in Figure S6). MEK1 showed interaction with PRO40 and MIK1, as seen in the TAP-MudPIT analysis, and with MAK1 only in the yeast two-hybrid analysis. However, we did not detect interactions between MEK1 and RHO1 or MEK1 and PKC1. Formation of homodimers was observed for PRO40, PKC1, MIK1, and MAK1. Figure 6C displays a schematic overview of signal transduction and protein-protein interactions in the PRO40-CWI complex.

To map PRO40 domains mediating interaction, we generated yeast two-hybrid vectors containing cDNA fragments of *pro40* (Figure 6A). PRO40 contains a WW domain, which is implicated in mediating protein-protein interactions [53], and several regions enriched for certain amino acids such as asparagine and glycine. Further, the PRO40 N-terminal half, enriched for glutamine and proline, is predicted to be highly disordered by IUPred [54], [55], as was also described for the *N. crassa* PRO40 homolog SOFT [56]. For yeast two-hybrid analysis, we generated five overlapping fragments PRO40a-e, containing different domains. As can be seen from Figure 6B, the N-terminal proline-rich and disordered PRO40 derivative, PRO40a, interacted with PRO40 itself as well as MEK1. PRO40c interacted with all full-length PRO40 interaction partners, namely PRO40, PKC1, MIK1, and MEK1. Since fragment PRO40c contains the WW domain, we hypothesized that binding of PRO40 to these interaction partners might be mediated by this domain. To test this hypothesis, we generated a modified PRO40, PRO40AAA (W575A, W598A, P601A), inserting mutations described to render the WW domain non-functional [57]. Surprisingly, PRO40AAA showed the same interactions as full-length PRO40 in a yeast two-hybrid assay (Figure 6B). PRO40AAA was further able to interact with itself.

We next generated MEK1 derivatives for yeast two-hybrid analysis (Figure 6A). As shown in Figure 6B, MEK1d comprising the kinase domain was sufficient for interaction with PRO40, MIK1, and MAK1. Moreover, interaction between MEK1 and MAK1 was accomplished via MEK1a and MEK1c, both comprising the proline-rich region. For interaction of MEK1 and PRO40, the MEK1 kinase domain (MEK1d) and either proline-rich PRO40a or central PRO40c were sufficient (Figure 6B). Figure 6D summarizes the results from interaction studies with PRO40 and MEK1 derivatives. Taken together, our data indicate that PRO40 connects the CWI MAPK module to its upstream activator PKC1.

### PRO40 is required for signaling via the CWI pathway

**Figure 7:**
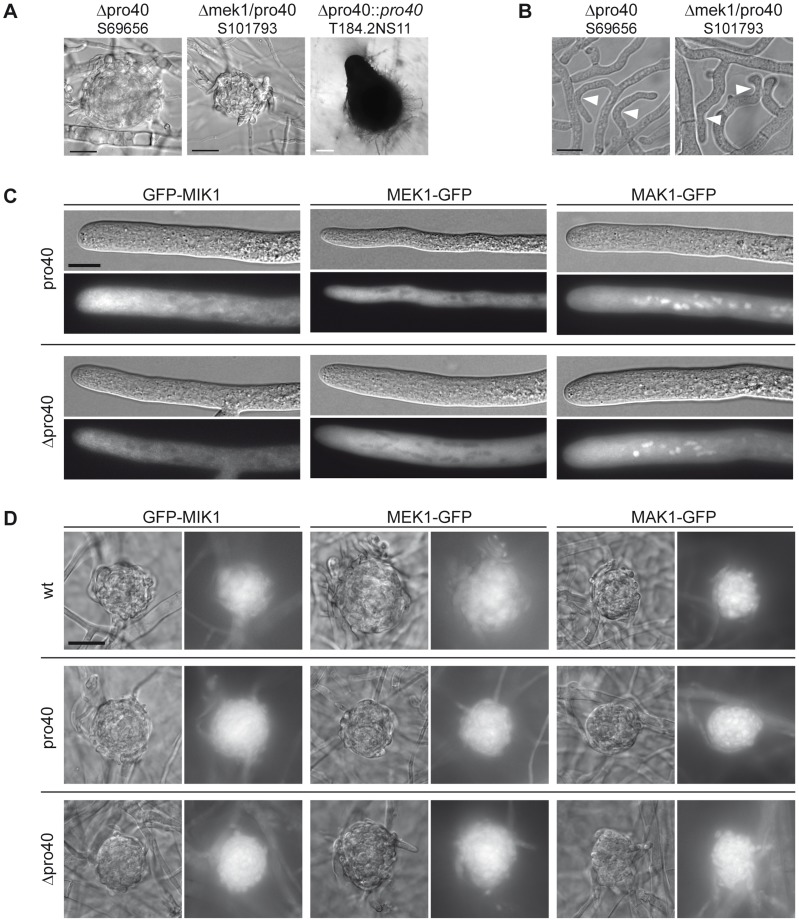
The Δmek1/pro40 double mutants shares phenotypic characteristics with Δmek1 and Δpro40. (A) Sexual development was assayed after 7 days of growth on BMM slides. Δpro40 and the Δmek1/pro40 double mutant generate only protoperithecia. White scale bar, 100 µm; black scale bar, 20 µm. (B) Δpro40 and the Δmek1/pro40 double mutant are unable to undergo hyphal fusion, although hyphae often grow in close contact (white arrowheads). Scale bar, 10 µm. (C) Localization of GFP-tagged MIK1, MEK1, and MAK1 in vegetative hyphae of the pro40 mutant and Δpro40. Scale bar, 10 µm. (D) Localization of GFP-tagged MIK1, MEK1, and MAK1 in three days old protoperithecia of the wildtype, the pro40 mutant, and the *pro40* deletion strain Δpro40. Scale bar, 20 µm.

To gain insight into the biological function of the MEK1-PRO40 interaction, we generated a Δmek1/pro40 double mutant. Single spore isolates from crosses of the single mutants were subjected to sequencing of the *pro40* ORF and Southern blot analysis (Figure S7). The Δmek1/pro40 double mutant was analyzed with regard to sexual development and hyphal fusion and showed the same developmental phenotype as the Δmek1 and Δpro40 single deletion strains (Figure 7A, B, compare Figure 2A, B). To test whether Δpro40 shares the stress response phenotype of the CWI kinase deletion strains, we performed growth tests on SWG ± CFW. As can be seen from Figure 3A, Δpro40 was less impaired in vegetative growth than the kinase deletion strains, and the growth reduction in the presence of CFW was also less distinctive. The Δmek1/pro40 double mutant was much more impaired than Δpro40 and showed the same growth defect as the Δmek1 single deletion strain. From these observations, we conclude that PRO40 does not play a major role in the cell wall stress response.

Since PRO40 interacts with three members of the CWI kinase pathway, we investigated whether the localization of MIK1, MEK1, and MAK1 was altered in the pro40 mutant or the Δpro40 deletion strain. Localization of the kinases in vegetative hyphae was identical in mutants and wildtype, with MIK1 and MEK1 residing in the cytoplasm and MAK1 localizing to the cytoplasm and the nucleus (Figure 7C, compare Figure 4A). Since PRO40 and the kinases are required for sexual development, we also investigated the localization of MIK1, MEK1, and MAK1 in protoperithecia of pro40, Δpro40, and wildtype. As illustrated in Figure 7D, GFP-MIK1 and MEK1-GFP showed a uniform distribution in protoperithecia of all investigated strains, most likely due to cytoplasmic localization. Similarly, the MAK1-GFP signal was found in the cytoplasm, and additionally in patches that resemble nuclei. In summary, the kinases showed the same localization in the presence and the absence of PRO40, in vegetative as well as in sexual tissues.

**Figure 8:**
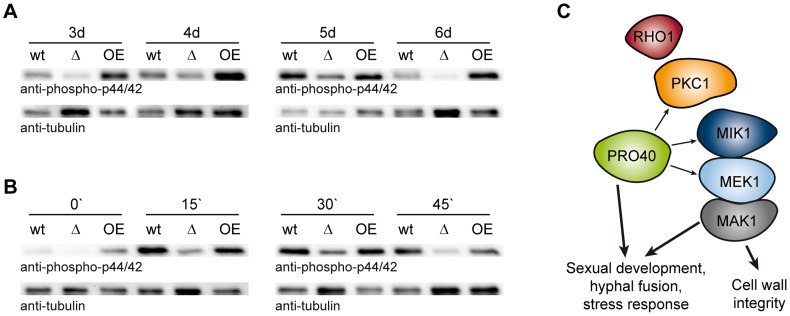
PRO40 is required for correct signaling via the CWI pathway. (A) Time course of MAK1 phosphorylation in *pro40* deletion (Δ; S69656) and overexpression strains (OE; T184.2NS11) in comparison to wildtype. Strains were grown for three to six days, and phosphorylated MAK1 was detected in a Western blot using an anti-phospho-p44/42 antibody. The signal for tubulin was used as internal standard. Representative immunoblots of two to four independent experiments with three technical replicates are shown. (B) Stress-induced MAK1 phosphorylation in *pro40* deletion (Δ; S69656) and overexpression strains (OE; T184.2NS11) in comparison to wildtype. Strains were grown for three days and subjected to 0.01% H_2_O_2_ for 0, 15, 30, and 45 minutes prior to harvesting. Phosphorylated MAK1 was detected using an anti-phospho-p44/42 antibody, and the signal for tubulin was used as internal standard. Representative immunoblots of three independent experiments with three technical replicates are shown. (C) Model of the scaffolding function of PRO40 for the CWI pathway. Details are discussed in the text.

To clarify the putative scaffolding role of PRO40 for the CWI pathway in more detail, we compared MAK1 phosphorylation levels in wildtype to MAK1 phosphorylation levels in Δpro40 and the *pro40* overexpression strain T182.4NS11 (Figure 8). First, we examined MAK1 phosphorylation during a developmental time course. As can be seen from Figure 8A, MAK1 activity is strongly reduced in Δpro40 in comparison to wildtype at all investigated time points. In contrast, MAK1 is hyper-phosphorylated in the *pro40* overexpression strain T184.2NS11. Second, we assayed MAK1 phosphorylation under stress conditions (Figure 8B). In the wildtype, MAK1 phosphorylation was strongly induced by H_2_O_2_. Again, MAK1 activity was strongly reduced in Δpro40; however, a response to 15 minutes H_2_O_2_ stress was still evident in the mutant. The *pro40* overexpression strain displayed MAK1 activity similar to wildtype. Thus, we concluded that PRO40 acts as a scaffold protein for CWI pathway components during sexual development, hyphal fusion, and stress response (Figure 8C).

## Discussion

In this study, we investigated the *S. macrospora* CWI kinase pathway and showed that the three kinases MIK1, MEK1, and MAK1 are required for the transition from protoperithecia to perithecia. Although the three deletion strains reach the same level of protoperithecia development, we observed that Δmak1 displayed significantly fewer protoperithecia than Δmik1 and Δmek1. This observation indicates that MAK1 obtains input not only from the upstream CWI kinases, but also from other pathways. Crosstalk between the CWI pathway and other stress response pathways has been observed in a number of fungi (reviewed by [58]). For example, the *N. crassa* PS and CWI pathways have both been described to control the cell wall stress response, hyphal fusion, and sexual development [10]. Here, we performed a large-scale analysis of MEK1 interactions in two different background strains. Our data establish a basis to functionally analyze further interaction partners of MEK1 for a regulatory role in CWI signaling.

In this study, we revealed the developmental protein PRO40 as a scaffold protein for the CWI pathway. *S. macrospora* PRO40 was previously identified by complementation of sterile mutant pro40, harboring a transition in the *pro40* gene that leads to an early translational stop [24]. Like its homolog SOFT from *N. crassa*, PRO40 is important for cell fusion [34], [35]. PRO40/SOFT homologs have further been shown to be important for cell fusion in *Alternaria brassicicola*, *Epichloë festucae*, *F. graminearum*, and *F. oxysporum* [46]–[48], [59]. In addition, PRO40/SOFT has been connected to pathogenicity in *A. brassicicola*, *F. graminearum*, and *F. oxysporum* and to symbiosis in *E. festucae* [46]–[48], [59]. Recently, *N. crassa* SOFT was shown to display an oscillatory localization at the tips of CATs approaching cell fusion, which alternates with the PS pathway MAPK MAK2 [60]. It was also proposed that SOFT and MAK2 act in two different signaling pathways with one sending and one receiving a yet unknown signal eventually leading to fusion of two CATs. Our data reveal that PRO40 of *S. macrospora* is a scaffold protein for another signaling pathway, namely the CWI pathway. Thus, a conceivable mechanism of PRO40 during CAT fusion is the regulation of the CWI kinases. All CWI kinase mutants of *N. crassa* have been described to be CAT fusion deficient [10], [61], and it should be highly elucidating to analyze the localization of the kinases during CAT fusion.

PRO40/SOFT homologs have been found in stress granules (*A. oryzae*), at septal pores in response to various stresses or hyphal injury (*A. oryzae*, *N. crassa*, *S. macrospora*), as well as associated with Woronin bodies (*S. macrospora*) [24], [62]–[64]. Woronin bodies are peroxisome-derived organelles found only in filamentous ascomycetes and contain a crystalline core assembled from the HEX1 protein [65]. An association of *S. macrospora* PRO40 with Woronin bodies was already found by co-localizing PRO40 with GFP-tagged HEX1 [24]. HEX1 was not identified as a significant PRO40 interaction partner in our experimental setup (Table S5); however, MEK1 was found to interact with HEX1 and Leashin, the Woronin body tether [66]. A connection of Woronin body function to the CWI pathway has already been observed in *A. oryzae*. There, PKC1 is required for HEX1 phosphorylation and subsequent HEX1 self-assembly [67]. The main function of Woronin bodies is the plugging of septal pores after hyphal injury, but they also play a role in plant infection and survival of nitrogen depletion in *M. grisea* [65], [68]. The Woronin body protein HEX1 is involved in asexual reproduction and virulence in *F. graminearum*, and *hex1* has been found to be regulated by PS MAPK MAK2 and downstream transcription factor PP-1, homologous to yeast Ste12p, in *N. crassa* [69], [70]. These findings are in agreement with a possible developmental role of Woronin bodies and the HEX1 protein.

By yeast two-hybrid analysis, we found two regions of PRO40 to be important for the observed interactions with CWI pathway components, namely the N-terminal proline-rich part and the central region encompassing the WW domain. Most interestingly, the N-terminal region of PRO40, including the proline-rich part, is also highly disordered. Protein disorder has been recognized as an important feature in signaling, since conformational fluctuations in disordered regions allow highly specific binding to multiple interaction partners in a regulated manner, thereby increasing functional capability [71]. Further analysis is needed to ascribe such versatile functions to PRO40 disordered regions. Another protein-protein interaction domain, the WW domain, has been found in PRO40. However, our yeast two-hybrid studies show that it is dispensable for interaction with PRO40, PKC1, MIK1, and MEK1. This indicates that PRO40 contains further unrecognized protein-protein interaction motifs within the region encompassing the WW domain.

Scaffold proteins are defined as proteins that not only bind to different signaling proteins, but that also attune signaling outputs [17], [72]. Although PRO40 does not affect the localization of MIK1, MEK1, and MAK1, it affects MAK1 phosphorylation, both during development and during stress response. Thus, PRO40 is a scaffold protein of the CWI pathway. We further attempted to address the question how PRO40 affects signaling via the CWI pathway by complementation analysis with constitutively active MAK1 and MEK1, inserting previously described mutations [73], [74]. However, these MAK1 and MEK1 versions were unable to reinstate wildtype morphology in the corresponding deletion mutants and thus were inept for further studies (our unpublished results). Since pro40 mutants did not display the same general growth defect as the CWI kinase mutants and were not as impaired as these mutants on media containing CFW, we conclude that PRO40 does not act as a scaffold of the CWI pathway during all CWI pathway functions. Recently, the presence of different CWI pathways has been suggested for *N. crassa*. There, different membrane sensors, WSC-1 and HAM-7, activate signaling via the CWI module, leading to cell wall stress response and hyphal fusion, respectively [75]. Our data strongly suggest that PRO40 acts as a scaffold protein for the CWI pathway during fungal development, hyphal fusion, and stress response.

In conclusion, we have identified PRO40 as a new scaffold protein of the highly conserved CWI pathway, linking the MAPK module to upstream activator PKC1. Collectively, our findings provide important insights into the mechanistic role of a fungal protein that has been implicated in sexual and asexual development, cell fusion, symbiosis, and pathogenicity in diverse fungal systems.

## Materials and Methods

### Strains and growth conditions

Cloning and propagation of recombinant plasmids was performed using standard laboratory conditions [76] and *Escherichia coli* strain XL1 Blue MRF' [77] as host for plasmid amplification. Alternatively to restriction-ligation-mediated cloning, recombinant plasmids were generated by homologous recombination in *S. cerevisiae* strains PJ69-4a, AH109 or Y187 [78], [79; Clontech, Palo Alto, CA, USA] as described previously [43], [80]. Recombinant yeasts were selected by prototrophy to leucine, tryptophan or uracil. Yeast experiments were carried out according to standard protocols (Clontech Yeast Protocol Handbook, PT3024-1), and plasmid isolation was performed as described by Bloemendal et al. [43].

The wildtype strain (S91327) of *S. macrospora* was obtained from our laboratory collection. Details for all *S. macrospora* strains used in this study are given in Table S8. Unless stated otherwise, standard growth conditions and DNA-mediated transformation were performed as described previously [81], [82]. Transformants were selected on medium containing either nourseothricin (50 µg/ml) or hygromycin B (80 U/ml). Sensitivity to Calcofluor White (CFW; Sigma Aldrich, St. Louis, MO, USA) was measured in 30 cm race tubes containing 15 ml solid SWG medium (derived from synthetic crossing medium according to Nowrousian et al. [83])±250 µg/ml CFW. For each strain, two race tubes were measured in each experiment and the growth front was marked every 24 hrs for 7 consecutive days. Preparation of DNA and Southern hybridization were performed as described [81].

### Generation of *S. macrospora* cDNA libraries

*S. macrospora* cDNA libraries SmI and SmII were generated using the Matchmaker Library Construction & Screening Kit (Clontech, Palo Alto, CA, USA). RNA was extracted from *S. macrospora* wildtype according to Pöggeler et al. [84] from 3 and 6 days old floating cultures (inducing sexual development) and 3 days old shaking cultures (repressing sexual development), and mRNA was isolated with the polyATtract mRNA isolation kit (Promega, Madison, WI, USA). For SmI, a mixture of 15 mRNA extractions and for SmII, a mixture of 7 mRNA extractions was used to generate cDNA according to the Matchmaker Library Construction & Screening Kit manual (PT3955-1, Clontech, Palo Alto, CA, USA). Each cDNA mixture was co-transformed with pGADT7-Rec into yeast strain AH109 and plated on SD medium lacking leucine. Colonies were harvested after 6 days of growth. Library titers were 4.3×10^7^/ml and 1.5×10^7^/ml for SmI and SmII, respectively.

### Cloning of yeast two-hybrid vectors

All plasmids and oligonucleotides used in this study are listed in Tables S9 and S10, respectively. For yeast two-hybrid analyses, PCR was performed on *S. macrospora* cDNA and PCR fragments cloned into pGBKT7 and pGADT7 as follows:

For RHO1, constitutively active (RHO1_CA) and constitutively inactive (RHO1_CI) versions were generated by inserting mutations G15V/C191S and E41I/C191S, respectively [85]. Specifically, a RHO1_CA fragment was generated from *S. macrospora* cDNA using primers rho1_CA-for/rho1_CA-rev and ligated *Eco*RI/*Bam*HI into pGADT7 and pGBKT7 to generate pA-RHO1_CA and pB-RHO1_CA, respectively. For pA-RHO1_CI, two PCR fragments generated with primer pairs rho1_CI-for x rho1_CIint-rev and rho1_CI-rev x rho1_CIint-for, were co-transformed into yeast with *Sma*I-digested pGADT7. pB-RHO1_CI was generated by ligating a 0.6 kb *Eco*RI/*Bam*HI fragment from pA-RHO1_CI into *Eco*RI/*Bam*HI-digested pGBKT7.

To generate pA-PKC1, yeast recombination was performed with *Sma*I-digested pGADT7 and three cDNA fragments amplified by PCR using primer pairs 4666-01-AD/4666-02, 4666-03/4666-04, and 4666-05/4666-06-AD. For pB-PKC1, a similar strategy was employed with *Sma*I-digested pGBKT7 and three PCR fragments produced with primer pairs 4666-01-BD/4666-02, 4666-03/4666-04, and 4666-05/4666-06-BD.

To generate pA-MIK1, yeast recombination was performed with *Eco*RI/*Bam*HI-digested pGADT7 and five PCR fragments produced with primer pairs 3673-1-AD/3673-2, 3673-3/3673-4, 3673-5/3673-6, 3673-7/3673-8, and 3673-9/3673-10-AD. For cloning of pB-MIK1, yeast recombination was again employed with *Sma*I-digested pGBKT7, two PCR fragments produced with primer pairs 3673-1-BD/3673-2 and 3673-9/3673-10-BD, and a 5254 bp *Nde*I/*Bam*HI fragment from pA-MIK1.

For *mek1* vectors, a 1458 bp *mek1* cDNA fragment was amplified with primers 6419-9/6419-10 and ligated *Eco*RI/*Bam*HI into pGBKT7 and pGADT7 to generate pB-MEK1_v01 and pA-MEK1_v01, respectively. For full-length cDNA vector pA-MEK1, pA-MEK1_v01 was digested with *Eco*RI and transformed into yeast together with a 425 bp *Bgl*II/*Bam*HI fragment of pA-MEK1a (see below). Likewise, pB-MEK1 was generated by yeast recombination using *Eco*RI-linearized pB-MEK1_v01and a 653 bp *Xho*I/*Bam*HI fragment of pB-MEK1a.

For *mak1* vectors, a PCR-fragment produced with primers HR-mak1-for/HR-mak1-rev as well as *Bam*HI-digested pGADT7 were transformed into yeast, generating pA-MAK1. pB-MAK1 was generated by ligation of a 1426 bp *Eco*RI/*Pst*I fragment from pA-MAK1 into *Eco*RI/*Pst*I-digested pGBKT7.

For pB-PRO40, full-length cDNA was amplified using primers Y2H-05/Y2H-06neu and ligated *Eco*RI/*Pst*I in pGBKT7. pA-PRO40 was generated by yeast recombination of two PCR fragments produced with primer pairs AD-40-for1/AD-40-rev1 and AD-40-for2/AD-40-rev2, and a 3961 bp *Eco*RI/*Pst*I fragment from pB-PRO40 into pGADT7/*Bam*HI.

To generate pA-MEK1a and pB-MEK1a, PCR was performed on *S. macrospora* cDNA using primer pair mek1_F1-fw/mek1_F1-rv, the PCR fragment subcloned into pDrive, cut *Eco*RI/*Bam*HI and ligated into *Eco*RI/*Bam*HI digested pGADT7 and pGBKT7, respectively. pA-MEK1b/pB-MEK1b, pA-MEK1c/pB-MEK1c and pA-MEK1d/pB-MEK1d were generated accordingly, using primer pairs mek1_F2-fw/mek1_F2-rv, mek1_F1-fw/mek1_F2-rv and mek1_F4-fw/6419-10, respectively.

To generate yeast two-hybrid vectors encoding PRO40 derivatives, five *pro40* fragments were amplified from cDNA and subsequently ligated into *Eco*RI and *Bam*HI sites of pGADT7 and pGBKT7. Primers used were Y2H-13/Y2H-07 for PRO40a, Y2H-08/Y2H-09 for PRO40b, Y2H-01/Y2H-12 for PRO40c, Y2H-10/Y2H-11 for PRO40d, and Y2H-03/Y2H-04 for PRO40e. pA-PRO40e was generated by ligating a 1007 bp *Eco*RI fragment from pB-PRO40e into the pGADT7 *Eco*RI site.

Vector pB-PRO40AAA encoding PRO40 with a mutated WW domain (PRO40 W575A, W598A, P601A) was generated by yeast recombination of a PCR fragment (primers 40-6/40-7) into *Sca*I-digested pB-PRO40. To generate pA-PRO40AAA, a 3961 bp *Eco*RI/*Pst*I-fragment from pB-PRO40AAA and a 10322 bp *Sal*I fragment from pA-40 were recombined in yeast.

### Yeast two-hybrid studies

The *S. macrospora pro40* cDNA was used as bait to screen both *S. macrospora* cDNA libraries for interacting proteins using the Matchmaker System (Clontech, Palo Alto, CA, USA). Yeast Y187 cells were transformed with pB-PRO40, mated with 1 ml cDNA library and plated on selective media (SD-trp-leu-ade and SD-trp-leu-his-ade). Colonies were re-inoculated on selective media lacking histidine or adenine and histidine. Growing yeast cells were subjected to two subsequent lacZ filter tests (Clontech Yeast Protocol Handbook, PT3024-1). 96 randomly chosen colonies showing reporter gene activity were chosen for PCR amplification of cDNA inserts using primer pair pAD-2/pAD-FPneu and PCR products were directly sequenced with primer pADfor96er. Quantitative measurements of β-galactosidase activity were carried out as described previously [86].

To test interactions between full-length proteins as well as derivatives of MEK1 and PRO40, strains carrying single plasmids were generated by electroporation [87] using matα strains (Y187, PJ69-4α) and mata strains (AH109, PJ69-4a) as recipients for BD and AD fusion constructs, respectively. Diploid strains were generated and tested for reporter gene expression as previously described [88]. For drop plating, yeast colonies were resuspended in 200 µl SD medium and 5 µl were spotted on SD supplemented with histidine and adenine as well as SD lacking histidine and adenine. Due to transactivation, pB-MEK1 was exchanged for pB-MEK1_v01, and pB-MEK1b, pB-MEKc and pA-RHO1_CA were omitted from the analysis.

### Generation of deletion strains

Deletion vectors for *mik1* and *mak1* were generated by yeast recombination as described [43]. For pKO-MIK1, 5′ (1000 bp) and 3′ (1000 bp) flanking regions of *mik1* were PCR-amplified using *S. macrospora* genomic DNA and primer pairs 3673-5fw/3673-5rv and 3673-3fw/3673-3rv, respectively. Flanking regions were transformed into yeast together with an *hph* cassette cut *Eco*RI from plasmid pDrivehph [89], and *Eco*RI/*Xho*I-linearized vector pRS426 [90]. Plasmid pKO-MAK1 was generated accordingly, using *mak1* 5′ (1000 bp) and 3′ (1039 bp) flanking regions amplified with primer pairs 5504-5fw/5504-5rv and 5504-3fwIT/5504-3rvIT, respectively.

To generate a *mek1* deletion, 5′ (832 bp) and 3′ (913 bp) flanking regions of *mek1* were PCR-amplified using primer pairs KO-mek-1/KO-mek-2 and KO-mek-3/KO-mek-4, respectively, and subcloned into pDrive. Due to annotation changes concerning *mek1* in genome version 02 of *S. macrospora* [25], a 5′-truncated version (*mek1_v01*, nt280–1858, encoding amino acids 35–519) was used as basis for generating *mek1* deletion and TAP vectors. The 5′ and 3′ regions were cut *Sna*BI/*Bam*HI and *Xba*I/*Apa*I and successively ligated into the corresponding sites of vector pDrive-Hyg (I. Godehardt and U. Kück, unpublished data). Linearized pKO-MIK1, pKO-MEK1 and pKO-MAK1 were transformed into *S. macrospora* Δku70 [32] and transformants were selected for by hygromycin resistance. Single-spore isolates in which *mik1*, *mek1* or *mak1* had been replaced by the *hph* cassette and which had the wildtype genetic background were obtained as described previously through crosses against spore color mutant fus or mutant pro40 [24], [25], [32].

### Cloning of vectors for complementation and localization

For pNTAP-mik1, yeast recombination was employed. Fragments used for transformation were *Bam*HI-digested pDS21 [91] and five PCR products generated with *S. macrospora* genomic DNA and primer pairs NTAP-mik-fw/3673-2, 3673-3/3673-4, 3673-5/3673-6, 3673-7/3673-8, and 3673-9/NTAP-mik-rv. pRSnat-gfp-mik1 was generated by amplification of *egfp* from pDS23 (M. Nowrousian, unpublished) using primers Pgpd_egfp_for/mik1_egfp_rev and subsequent recombination in yeast with *Hin*dIII-digested pNTAP-mik1. For pGFP-MIK1_NA, 5′ and 3′ *mik1* sequences were amplified from *S. macrospora* genomic DNA with primer pairs 3673-5fw/3673-5rv-gfp and 3673-11/3673-3rv, respectively, and subsequently recombined into pRSnat-gfp-mik1, replacing the *gpd* promoter and *trpC* terminator.

For complementation and TAP, *mek1* was amplified from genomic DNA with primers mek1-BamHI-fw/NTAP-mek-BamHI-rv, subcloned into pDrive, cut with *Bam*HI and cloned into *Bam*HI-digested pDS21 [91], generating pNTAP-MEK1. Vector pRSnat-mek1-gfp_V3 was generated by amplifying *mek1* from *S. macrospora* genomic DNA with primer pair Pgpd-mek1_V3/gfp-mek-rev and recombination into linearized pDS23 in yeast. For pMEK1-GFP_NA, 5′ and 3′ *mek1* sequences were amplified from *S. macrospora* genomic DNA with primer pairs 2183-5fw_IT/mek1_F1-rv and 2183-3fw-gfp/2183-3rv_IT, respectively, and transformed into yeast together with a 2.8 kb *Pvu*II-*Spe*I fragment from pRSnat-mek1-gfp_V3 and linearized pRSnat [92].

A *mak1* complementation vector was constructed by amplifying *mak1* using primers Pxyl-mak-for/NTAP-MAK-rv, and recombining the PCR fragment into *Not*I/*Bam*HI-digested pNpX-GFP [38], yielding pNpX-MAK1. Vector pRSnat-mak1-gfp was generated by amplifying *mak1* from genomic DNA with primer pair CTAP-mak1-fw/GFP-mak1-rv and transforming the PCR fragment in yeast together with *Hin*dIII-linearized pDS23. For pMAK1-GFP_NA, 5′ (5504-5fw/GFP-mak1-rv) and 3′ (5504-3fw-gfp/5504-3rv_IT) *mak1* sequences were amplified and transformed in yeast together with a 0.8 kb *Bam*HI fragment from pRSnat-mak1-gfp and linearized pRSnat.

### Affinity purification

To search for PRO40 interaction partners, pC-FLAG-PRO40 [24] was transformed into Δpro40 and single spore isolate T184.2NS11 was used for further analysis. For FLAG-AP, dried mycelium was ground in liquid nitrogen, suspended in FLAG extraction buffer (50 mM Tris-HCl pH 7.4, 250 mM NaCl, 10% glycerol, 0.05% NP-40, 1 mM PMSF, 0.2% protease inhibitor cocktail IV (Calbiochem), 1 mM benzamidine, 1 µg/ml leupeptin) and centrifuged for 30 min at 16000 rpm. 50 ml crude protein extract was incubated with 300 µl anti-FLAG M2 affinity gel (A2220, Sigma Aldrich, St. Louis, MO, USA) overnight at 4°C on a rotator. Bound complexes were collected by centrifugation and washed twice in 45 ml and once in 1 ml cold washing buffer (50 mM Tris-HCl pH 7.4, 150 mM NaCl, 0.05% NP-40, 1 mM PMSF, 1 mM benzamidine, 1 µg/ml leupeptin) with rotation at 10 min intervals. The affinity gel was transferred to a 1.5-ml centrifuge tube and incubated in 500 µl of cold washing buffer containing 2 µl protease inhibitor cocktail IV (Calbiochem) and 0.5 mg/ml 3× FLAG peptide (F4799, Sigma Aldrich, St. Louis, MO, USA) for 6 hr at 4°C on a rotator. After centrifugation, the supernatant was transferred to a new 1.5-ml tube, the gel briefly washed in 500 µl cold washing buffer, centrifuged and the supernatant was combined with the first supernatant. Purified complexes were subjected to trichloroacetic acid precipitation and directly used for mass spectrometry.

For TAP analysis, pNTAP-mek1 was transformed into *S. macrospora* Δmek1 and Δpro40, and transformants were selected on media with nourseothricin. Primary transformants expressing NTAP-MEK1 were used for single spore isolation. For protein extraction, *S. macrospora* strains E292 (Δmek1::NTAP-MEK1) and E2544 (Δpro40::NTAP-MEK1) were grown in P-flasks with BMM liquid medium for 3 d at 27°C. TAP analysis was performed as described previously [43].

### Mass spectrometry

Tryptic digestion of proteins and MudPIT analysis [44], [45] were performed as described previously [43] using an Orbitrap Velos ion trap mass spectrometer coupled to an Accela quaternary U-HPLC pump (Thermo Fisher Scientific). Proteome Discoverer software version 1.2 was used for MS/MS data interpretation, and data were searched against the *S. macrospora* database (smacrosporapep_v4_110909) with tryptic peptides, mass accuracy of 10 ppm, fragment ion tolerance of 0.8 Da, and with oxidation of methionine as variable modification allowing 4 missed cleavage sites. All accepted results had a high peptide confidence with a score of 10. Proteins identifies with at least two different peptides in at least two of three to four independent experiments were considered for further analysis.

To identify contaminants in TAP-MudPIT data, we used an extended background list from Bloemendal et al. [43] (Table S6). For validation of PRO40-FLAG-MS data, we performed FLAG-AP experiments with wildtype protein extract as control. Since these experiments yielded a large number of proteins, a previously described quantification approach was employed to evaluate proteins that were identified with at least two different peptides in 2–3 PRO40-FLAG-AP experiments (Table S3), but also in wildtype control experiments (Table S4) [93], [94]. This procedure was necessary, because the PRO40 bait protein was identified in one of the control experiments. Therefore, spectral counts for each identified protein were first divided by the sum of spectral counts for each MS run (PRO40_1, 9559; PRO40_2, 5010; PRO40_3, 7999; wt_1, 5633; wt_2, 5901). Then, values for each experiment type were added and used to calculate a ratio between PRO40 and wildtype control data (Table S5). 79 proteins showing a ratio ≥2 were considered significant hits. From these proteins, PRO40 (ratio 18.63) and MEK1 (ratio 2.15) were verified as direct PRO40 binding proteins by yeast two-hybrid analysis, and RHO1 (ratio 2.23) was verified as indirect PRO40 binding protein via the interaction with PKC1, showing the applicability of this approach.

### Immunodetection

Immunodetection of TAP-tagged proteins was performed as described using a polyclonal anti-calmodulin binding peptide antibody (1∶2000, Merck Millipore, Billerica, MA, USA) and an anti-rabbit HRP-linked secondary antibody (Cell Signaling Technology, Danvers, MA, USA) [43]. FLAG-PRO40 was detected as described [24] using a monoclonal anti-FLAG antibody (1∶2000, Sigma Aldrich, St. Louis, MO, USA) and an anti-mouse HRP-linked secondary antibody (Cell Signaling Technology, Danvers, MA, USA).

### Phosphorylation studies

For analysis of MAK1 phosphorylation status, strains were pre-cultured in liquid BMM for 2 days at 27°C. Three standardized inoculates were transferred into liquid HEPES (50 mM) -buffered BMM and cultivated for an additional three to six days at 27°C and 30 rpm. For induction of cell wall stress, cultures were subjected to 0.01% H_2_O_2_ for 15, 30, or 45 minutes. Mycelia were harvested by filtration, ground in liquid nitrogen, and resuspended in FLAG extraction buffer with phosphatase inhibitors (1% Phosphatase-Inhibitor-Cocktails II and III, Sigma Aldrich, St. Louis, MO, USA). After centrifugation at 15000 rpm for 30 min, equal amounts of total protein were subjected to SDS PAGE and Western Blotting according to standard protocols [76]. Phosphorylated MAK1 was detected using a polyclonal anti-phospho-p44/42 antibody (Cell Signaling Technology, Inc., USA) and an anti-rabbit HRP-linked secondary antibody (Cell Signaling Technology, Danvers, MA, USA) according to the manufacturer's protocol. Chemiluminescence was detected using a ChemidocXRS system (Biorad) and Clarity Western ECL substrate (Biorad). For an internal standard, an anti-α-tubulin antibody (Sigma Aldrich, St. Louis, MO, USA, T9026) was used in combination with an anti-mouse HRP-linked secondary antibody (Cell Signaling Technology, Danvers, MA, USA).

### Microscopic investigations

Microscopy was performed with an AxioImager microscope (Zeiss, Jena, Germany). For characterization of sexual development by DIC microscopy, strains were grown on BMM-coated glass slides for 2–7 days as described previously [24]. Hyphal fusion was investigated in 2 days old cultures grown on cellophane-covered MMS plates as described [43]. Localization of fluorescently labeled proteins in vegetative hyphae was investigated on BMM-covered glass slides as described previously [24]. For fluorescence microscopy of protoperithecia, strains were grown on cellophane-covered BMM plates for 3 days, and pieces of cellophane were fixed in 0.2% formaldehyde in PBS (58 mM Na_2_HPO_4_, 17 mM NaH_2_PO_4_, 68 mM NaCl; pH 7.4). Fluorescence was observed using filter sets (Chroma Technology) 41017 (HQ470/40, HQ525/50, Q495lp) or 49002 (ET470/40×, ET525/50m, T495lpxr) for EGFP and filter set 49008 (ET560/40×, ET630/75m, T585lp) for tdTomato.

### DNA preparation, Illumina sequencing and mapping

Mutant pro30 from our laboratory collection was back-crossed several times to wildtype or brown-spored fus [25] and finally crossed to fus (Figure S8). DNA was extracted from 40 sterile progeny as described previously [25]. 40 fertile strains were collected from three crosses of mutants pro30, pro32, and pro34 to fus (Figure S8). Mutants pro32 and pro34 are described elsewhere [82; Teichert and Kück, unpublished]. 5 µg of pooled genomic DNA for pro30 and wt, respectively, was subjected to 50 bp paired-end Illumina/Solexa sequencing with a HiSeq2000 at GATC Biotech (Konstanz, Germany). Cleaning of raw data, mapping to the *S. macrospora* reference genome [5], [41], and analysis of sequence variants was performed as described [25] using the Burrows Wheeler Alignment tool [95], SAMtools [96] and custom-made Perl scripts, with minor modifications (Text S1). Genome sequencing data have been deposited at the sequence read archive (SRA; acc. no. SRX483430 and SRR1046323 for pro30 and wildtype (wt_3) [82], respectively).

## Supporting Information

Figure S1 The *mik1* gene is mutated in the pro30 mutant. (A) Structure of the *S. macrospora mik1* gene with introns (white boxes). The deduced MIK1 protein structure is displayed above the gene structure. The C904T mutation is indicated. Domains are displayed as in Figure 6A. (B) Sequencing of the *mik1* gene in 20 ascospore lines from a pro30 to fus cross. Sterile strains display the C to T transition present in pro30, while fertile strains display the wildtype CAG codon. The transition leads to an exchange of the CAG codon to a TAG stop codon (boxed).
(TIF)

Figure S2 Generation of Δmik1 deletion strains. (A) Schematic representation of the *mik1* genomic locus in wildtype and deletion strains. ORFs are displayed as grey arrows, with introns marked as white boxes. Flanking sequences used for homologous integration of knockout constructs are shown as thick black lines. Black arrows represent primers used for PCR; size of PCR products is given on the thin black lines depicting the PCR products. Probes used for Southern hybridization are shown as grey bars, with grey lines indicating the size of expected signals. Restriction enzymes used for digestion of genomic DNA are depicted in grey. Not drawn to scale. (B) PCR analysis of Δmik1 strains S113578 and S113494, as well as wildtype (WT). NK, negative control. (C) Southern analysis of strains from (B) with *hph* and *mik1* probes as illustrated in (A).
(TIF)

Figure S3 Generation of Δmek1 deletion strains. (A) Schematic representation of the *mek1* genomic locus in wildtype and deletion strains. ORFs are displayed as grey arrows, with introns marked as white boxes. Flanking sequences used for homologous integration of knockout constructs are shown as thick black lines. Black arrows represent primers used for PCR; size of PCR products is given on the thin black lines depicting the PCR products. Probes used for Southern hybridization are shown as grey bars, with grey lines indicating the size of expected signals. Restriction enzymes used for digestion of genomic DNA are depicted in grey. Not drawn to scale. (B) PCR analysis of Δmek1 strains S101796 and S101767, as well as wildtype (WT). NK, negative control. (C) Southern analysis of strains from (B) with *hph* and *mek1* probes as illustrated in (A).
(TIF)

Figure S4 Generation of Δmak1 deletion strains. (A) Schematic representation of the *mak1* genomic locus in wildtype and deletion strains. ORFs are displayed as grey arrows, with introns marked as white boxes. Flanking sequences used for homologous integration of knockout constructs are shown as thick black lines. Black arrows represent primers used for PCR; size of PCR products is given on the thin black lines depicting the PCR products. Probes used for Southern hybridization are shown as grey bars, with grey lines indicating the size of expected signals. Restriction enzymes used for digestion of genomic DNA are depicted in grey. Not drawn to scale. (B) PCR analysis of Δmak1 strains R8501 and R8533, as well as wildtype (WT). NK, negative control. (C) Southern analysis of strains from (B) with *hph* and *mak1* probes as illustrated in (A).
(TIF)

Figure S5 Affinity purification of PRO40 and MEK1. (A) SDS-PAGE and immunodetection of PRO40-FLAG. Aliquots of crude extract, washing steps, FLAG beads and elution were analyzed by immunodetection with an anti-FLAG antibody. PRO40 is strongly enriched in the elution. (B), (C), SDS-PAGE and immunodetection of NTAP-MEK1. Crude extracts of a strain E292 (B) and E2544 (C) expressing NTAP-MEK1 show the total soluble protein before purification. Aliquots of flowthrough 1 and washing step 1 (IgG beads), flowthrough 2 and washing step 2 (CBP beads), and the elution were analyzed. Immunodetection with an anti-CBP antibody shows NTAP-MEK1 with a size of 76 kDa in the crude extracts and washing steps 1 and CBP-MEK1 with a size of 57 kDa in the elution.
(TIF)

Figure S6 Growth control of yeast colonies from yeast two-hybrid assays. This figure is related to Figure 6B. Colonies were plated on SD-leu-trp.
(TIF)

Figure S7 Generation of Δmek1/pro40 double mutants. (A) Sequence analysis of strains from a Δku70/Δmek1 to pro40 cross. Strains S101796 and S101767 show the wildtype (WT) sequence, whereas strains S100793 and S101820 show the pro40 mutation. (B) PCR analysis of Δmek1/pro40 strains S101793 and S101820 to verify the *mek1* deletion. WT, wildtype; NK, negative control. Primers are depicted in Figure S3A.
(TIF)

Figure S8 Crossing history of strains for genome sequencing. (A) Crossing history of mutant pro30. Strains were backcrossed to wildtype (wt) or spore color mutant fus [25], which is fertile but produces light-brown instead of black spores. (B) Strategy for whole-genome sequencing of pooled DNA from mutants and wildtype. Mutants pro30, pro32, and pro34 were crossed to spore color mutant fus. Single spore isolates derived from black and light-brown ascospores were screened for fertility and color. For sample pro30, 40 single spore isolates with a sterile phenotype were chosen. For re-sequencing of the wildtype, 40 isolates with a fertile phenotype were chosen from the three crosses. The pooled DNA from 40 single spore isolates for each genotype (pro30 and wt_3) was used for sequencing.
(TIF)

Figure S9 Alignment of MIK1 homologs from *S. macrospora*, *N. crassa*, and *S. cerevisiae*. Alignments were generated with ClustalW (http://www.genome.jp/tools/clustalw/) and edited in GeneDoc. Identity to the *S. macrospora* proteins is given in percent. Sm_MIK1, CCC09641.1; Nc_MIK-1, XP_959647.2; Sc_Bck1p, EWG95039.1.
(TIF)

Figure S10 Alignment of MEK1 homologs from *S. macrospora*, *N. crassa*, and *S. cerevisiae*. Alignments were generated with ClustalW (http://www.genome.jp/tools/clustalw/) and edited in GeneDoc. Identity to the *S. macrospora* proteins is given in percent. Sm_MEK1, CCC11961.1; Nc_MEK-1, XP_957310.2; Sc_Mkk1p, EWG93186.1; Sc_Mkk2p, EWG93010.1.
(TIF)

Figure S11 Alignment of MAK1 homologs from *S. macrospora*, *N. crassa*, and *S. cerevisiae*. Alignments were generated with ClustalW (http://www.genome.jp/tools/clustalw/) and edited in GeneDoc. Identity to the *S. macrospora* proteins is given in percent. Sm_MAK1, CCC12327.1; Nc_MAK-1, EAA28804.2; Sc_Mpk1p, AAB26249.1.
(TIF)

Table S1 Summary of sequence reads and small variants from genome sequencing of pro30 and wildtype.
(PDF)

Table S2 List of MEK1 interaction partners identified by tandem affinity purification and mass spectrometry.
(XLSX)

Table S3 List of PRO40 interaction partners identified by FLAG affinity purification and mass spectrometry.
(XLSX)

Table S4 List of proteins identified in control experiments by FLAG affinity purification and mass spectrometry.
(XLSX)

Table S5 Evaluation of significance of PRO40 interaction partners.
(XLSX)

Table S6 List of known contaminants from affinity purification experiments.
(XLSX)

Table S7 List of putative PRO40 interaction partners identified by yeast two-hybrid screens.
(PDF)

Table S8 Strains used in this study.
(PDF)

Table S9 Plasmids used in this study.
(PDF)

Table S10 Oligonucleotides used in this study.
(PDF)

Text S1 Supplementary methods.
(PDF)
