# Supplementary figures and images for: PRO40 Is a Scaffold Protein of the Cell Wall Integrity Pathway, Linking the MAP Kinase Module to the Upstream Activator Protein Kinase C
Source: PLoS Genet. 2014 Sep 4;10(9):e1004582. doi: 10.1371/journal.pgen.1004582 (PMC4154660; doi:10.1371/journal.pgen.1004582)

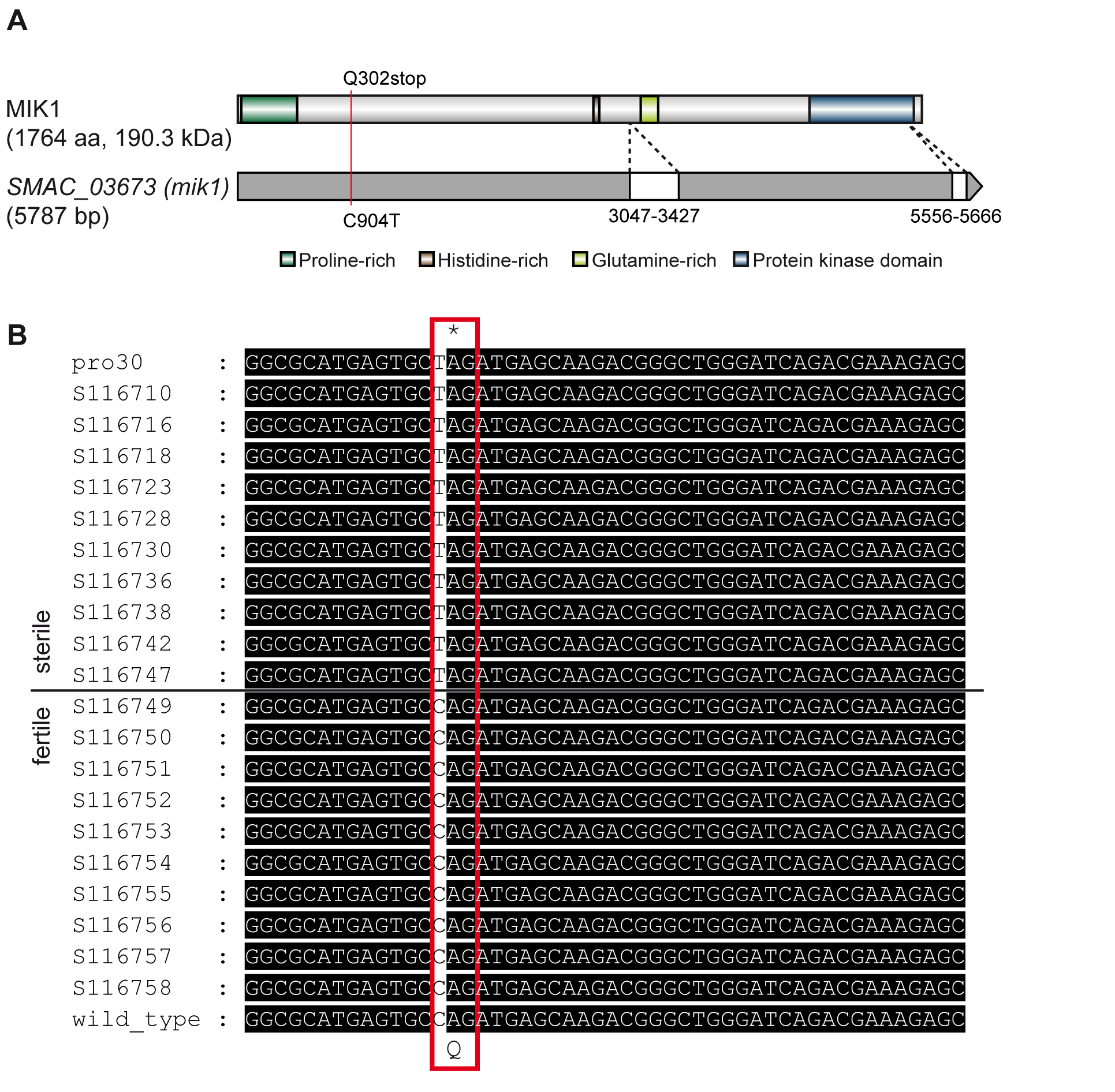

Supplement: Figure S1 — The mik1 gene is mutated in the pro30 mutant. (A) Structure of the S. macrospora mik1 gene with introns (white boxes). The deduced MIK1 protein structure is displayed above the gene structure. The C904T mutation is indicated. Domains are displayed as in Figure 6A. (B) Sequencing of the mik1 gene in 20 ascospore lines from a pro30 to fus cross. Sterile strains display the C to T transition present in pro30, while fertile strains display the wildtype CAG codon. The transition leads to an exchange of the CAG codon to a TAG stop codon (boxed). (TIF) [file pgen.1004582.s001.tif]

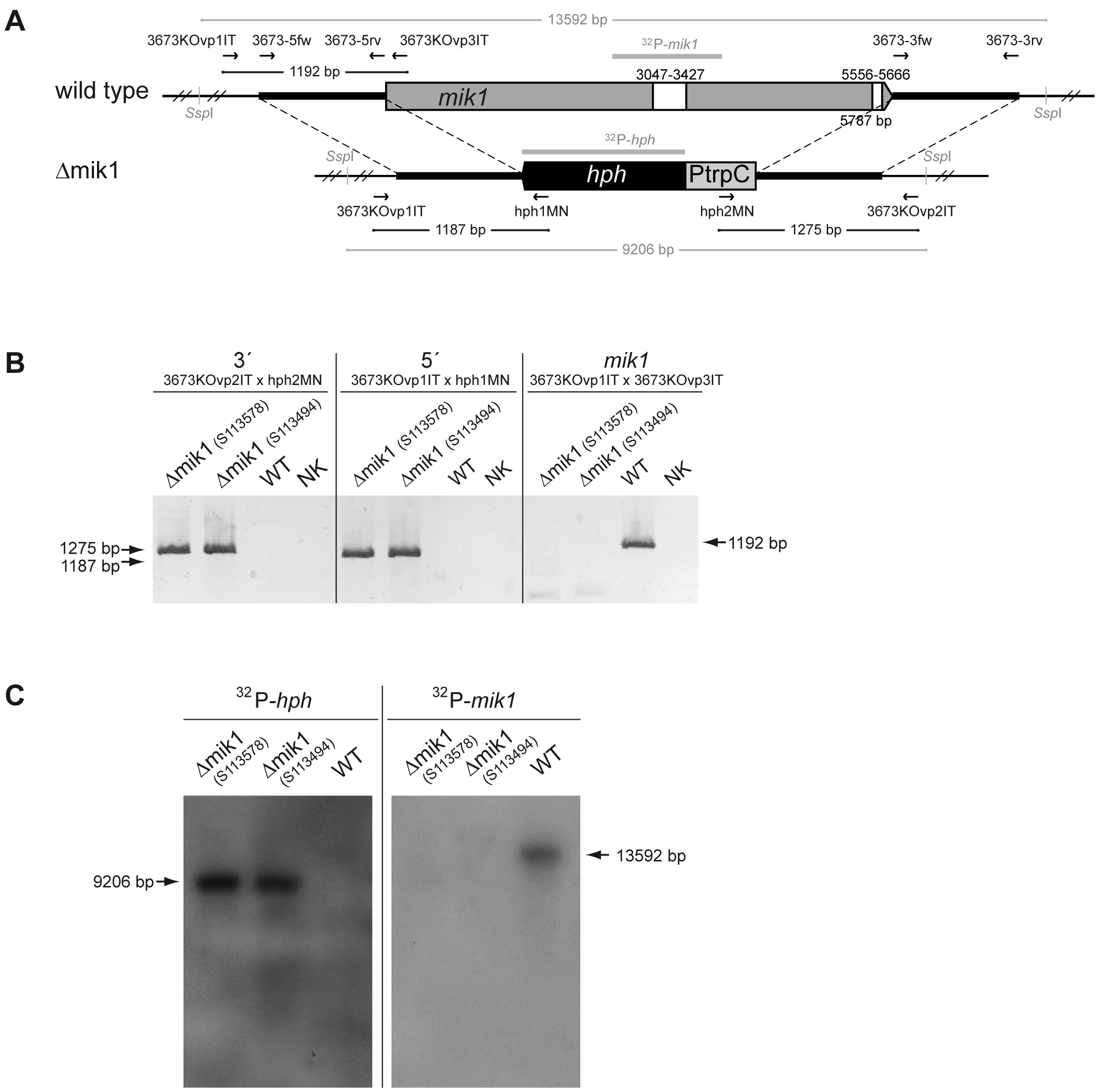

Supplement: Figure S2 — Generation of Δmik1 deletion strains. (A) Schematic representation of the mik1 genomic locus in wildtype and deletion strains. ORFs are displayed as grey arrows, with introns marked as white boxes. Flanking sequences used for homologous integration of knockout constructs are shown as thick black lines. Black arrows represent primers used for PCR; size of PCR products is given on the thin black lines depicting the PCR products. Probes used for Southern hybridization are shown as grey bars, with grey lines indicating the size of expected signals. Restriction enzymes used for digestion of genomic DNA are depicted in grey. Not drawn to scale. (B) PCR analysis of Δmik1 strains S113578 and S113494, as well as wildtype (WT). NK, negative control. (C) Southern analysis of strains from (B) with hph and mik1 probes as illustrated in (A). (TIF) [file pgen.1004582.s002.tif]

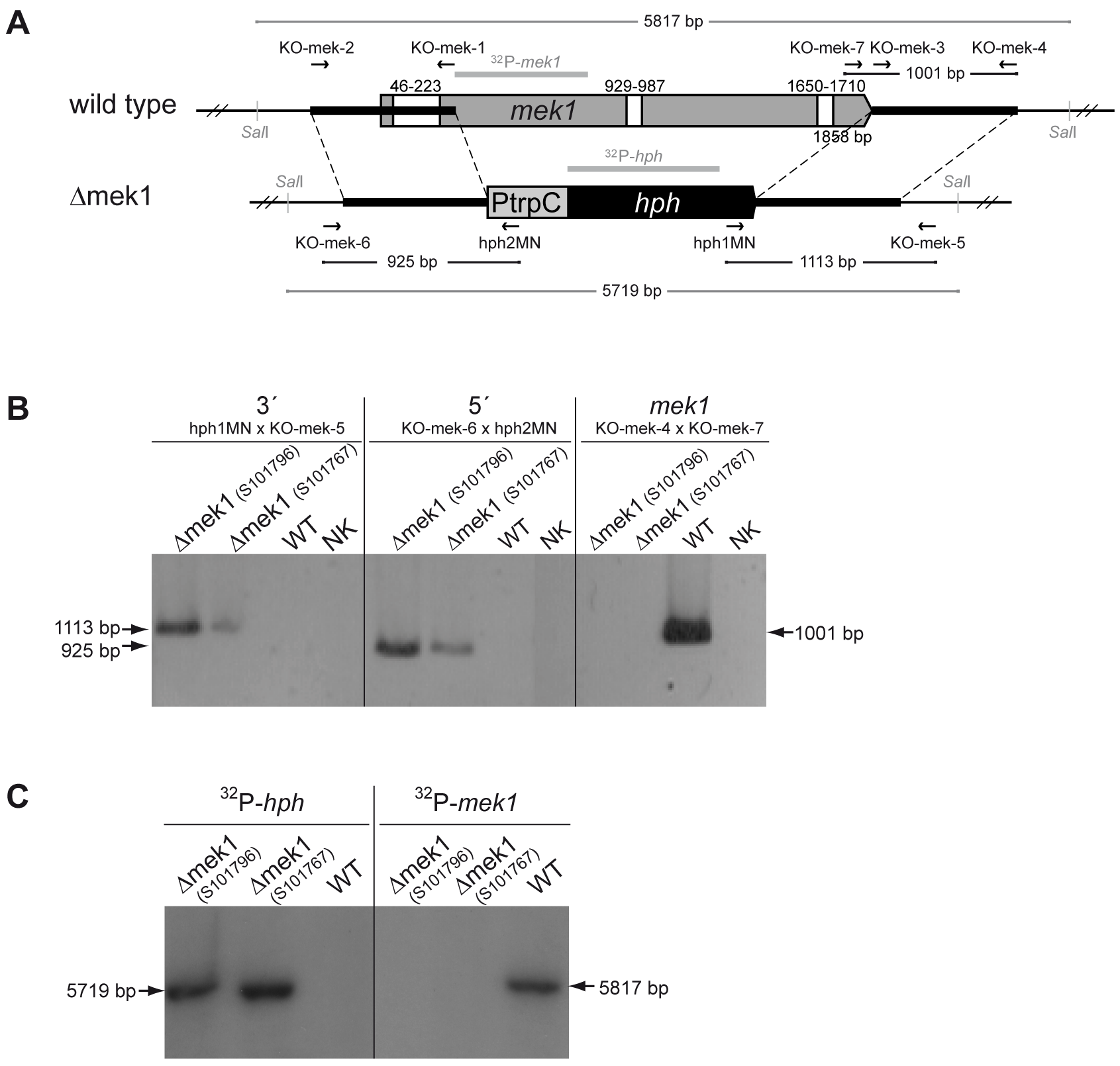

Supplement: Figure S3 — Generation of Δmek1 deletion strains. (A) Schematic representation of the mek1 genomic locus in wildtype and deletion strains. ORFs are displayed as grey arrows, with introns marked as white boxes. Flanking sequences used for homologous integration of knockout constructs are shown as thick black lines. Black arrows represent primers used for PCR; size of PCR products is given on the thin black lines depicting the PCR products. Probes used for Southern hybridization are shown as grey bars, with grey lines indicating the size of expected signals. Restriction enzymes used for digestion of genomic DNA are depicted in grey. Not drawn to scale. (B) PCR analysis of Δmek1 strains S101796 and S101767, as well as wildtype (WT). NK, negative control. (C) Southern analysis of strains from (B) with hph and mek1 probes as illustrated in (A). (TIF) [file pgen.1004582.s003.tif]

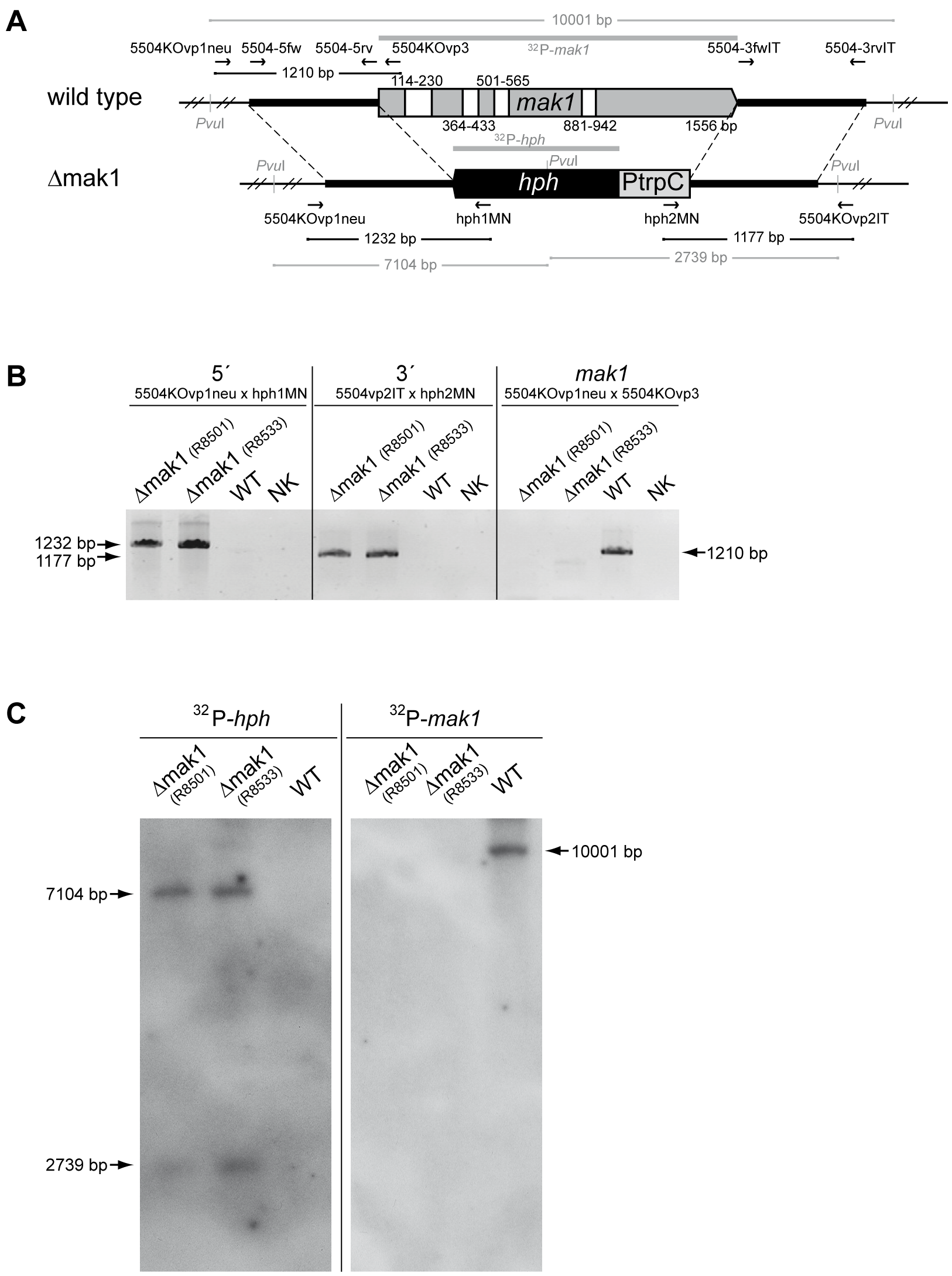

Supplement: Figure S4 — Generation of Δmak1 deletion strains. (A) Schematic representation of the mak1 genomic locus in wildtype and deletion strains. ORFs are displayed as grey arrows, with introns marked as white boxes. Flanking sequences used for homologous integration of knockout constructs are shown as thick black lines. Black arrows represent primers used for PCR; size of PCR products is given on the thin black lines depicting the PCR products. Probes used for Southern hybridization are shown as grey bars, with grey lines indicating the size of expected signals. Restriction enzymes used for digestion of genomic DNA are depicted in grey. Not drawn to scale. (B) PCR analysis of Δmak1 strains R8501 and R8533, as well as wildtype (WT). NK, negative control. (C) Southern analysis of strains from (B) with hph and mak1 probes as illustrated in (A). (TIF) [file pgen.1004582.s004.tif]

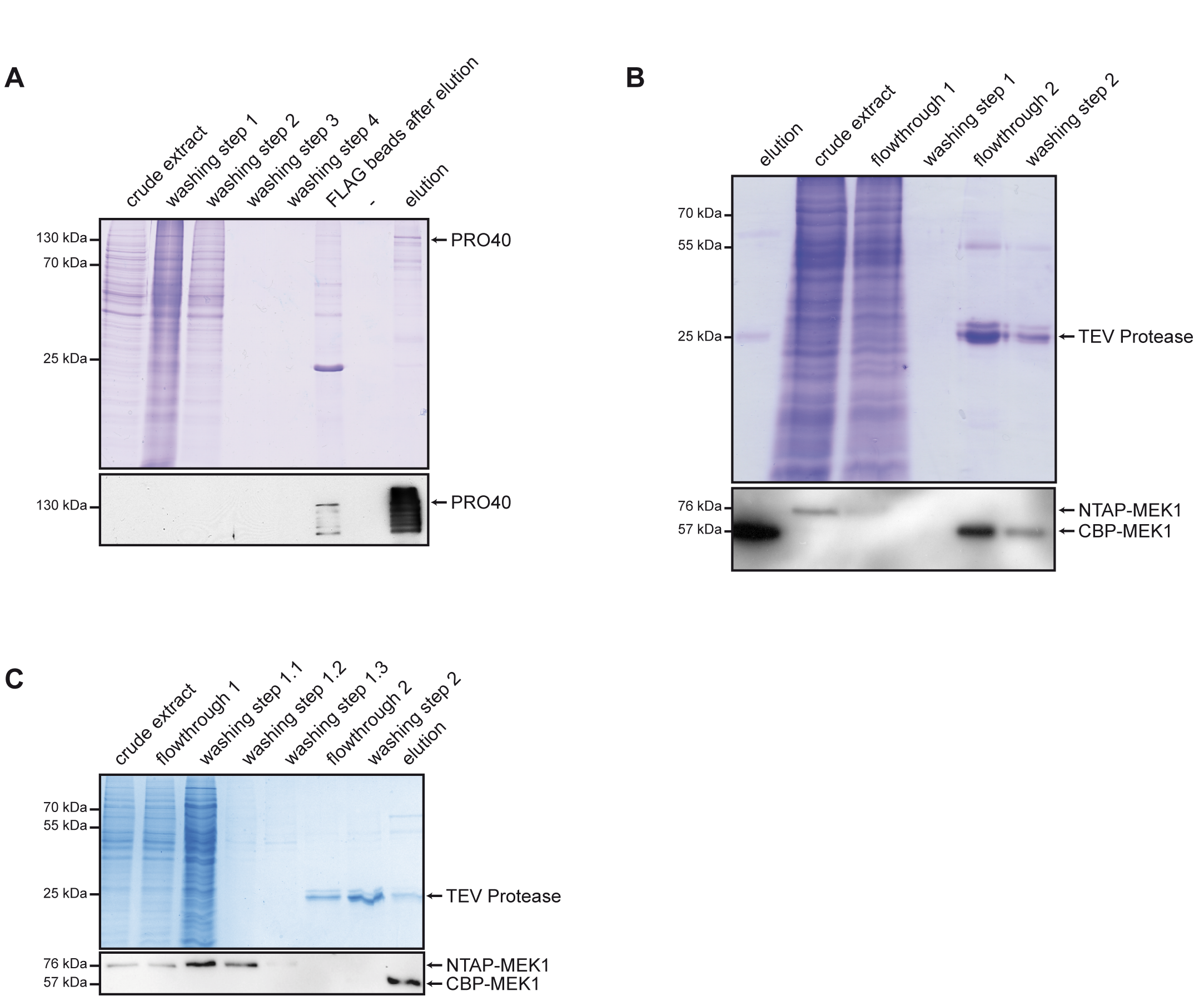

Supplement: Figure S5 — Affinity purification of PRO40 and MEK1. (A) SDS-PAGE and immunodetection of PRO40-FLAG. Aliquots of crude extract, washing steps, FLAG beads and elution were analyzed by immunodetection with an anti-FLAG antibody. PRO40 is strongly enriched in the elution. (B), (C), SDS-PAGE and immunodetection of NTAP-MEK1. Crude extracts of a strain E292 (B) and E2544 (C) expressing NTAP-MEK1 show the total soluble protein before purification. Aliquots of flowthrough 1 and washing step 1 (IgG beads), flowthrough 2 and washing step 2 (CBP beads), and the elution were analyzed. Immunodetection with an anti-CBP antibody shows NTAP-MEK1 with a size of 76 kDa in the crude extracts and washing steps 1 and CBP-MEK1 with a size of 57 kDa in the elution. (TIF) [file pgen.1004582.s005.tif]

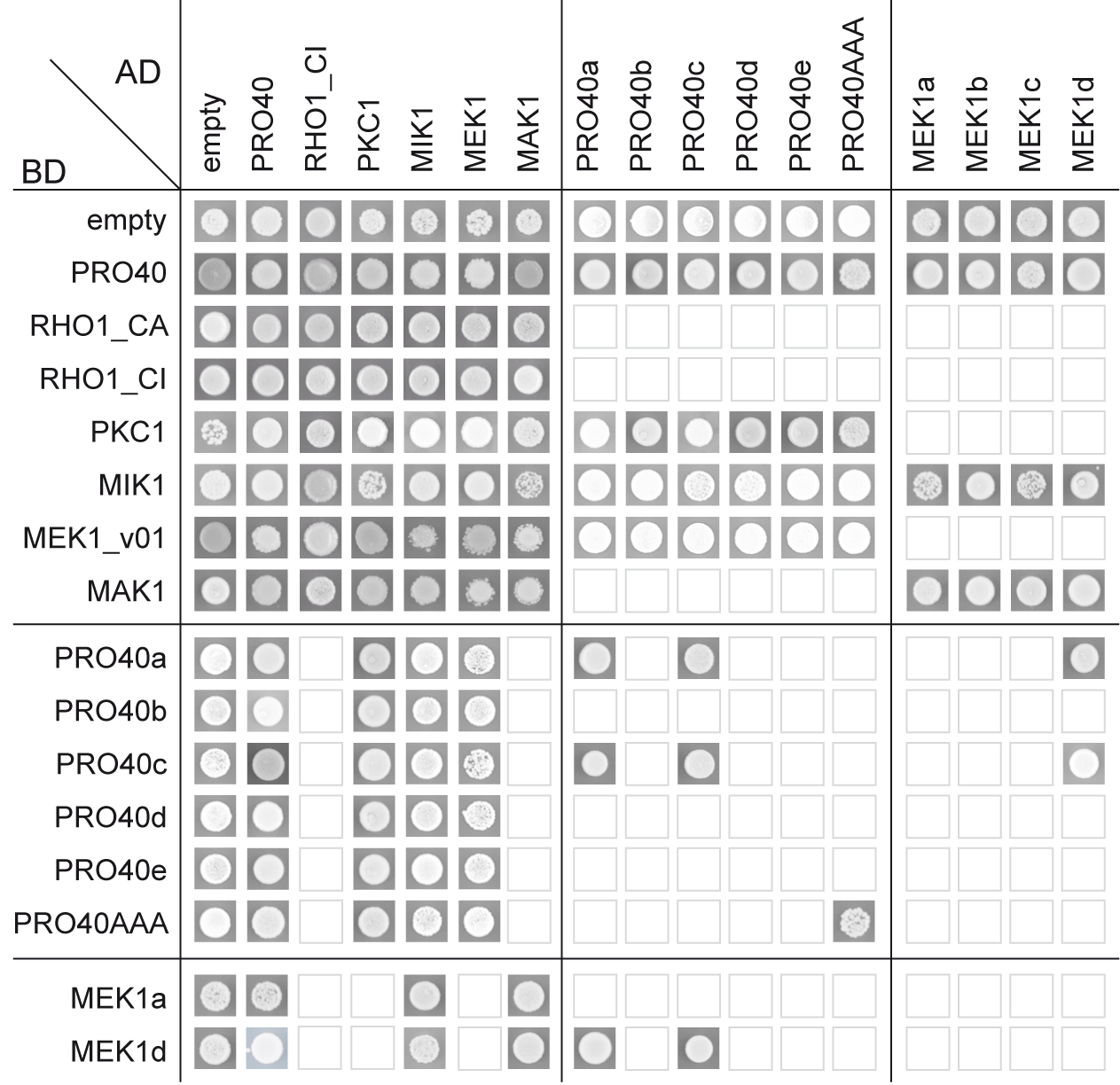

Supplement: Figure S6 — Growth control of yeast colonies from yeast two-hybrid assays. This figure is related to Figure 6B. Colonies were plated on SD-leu-trp. (TIF) [file pgen.1004582.s006.tif]

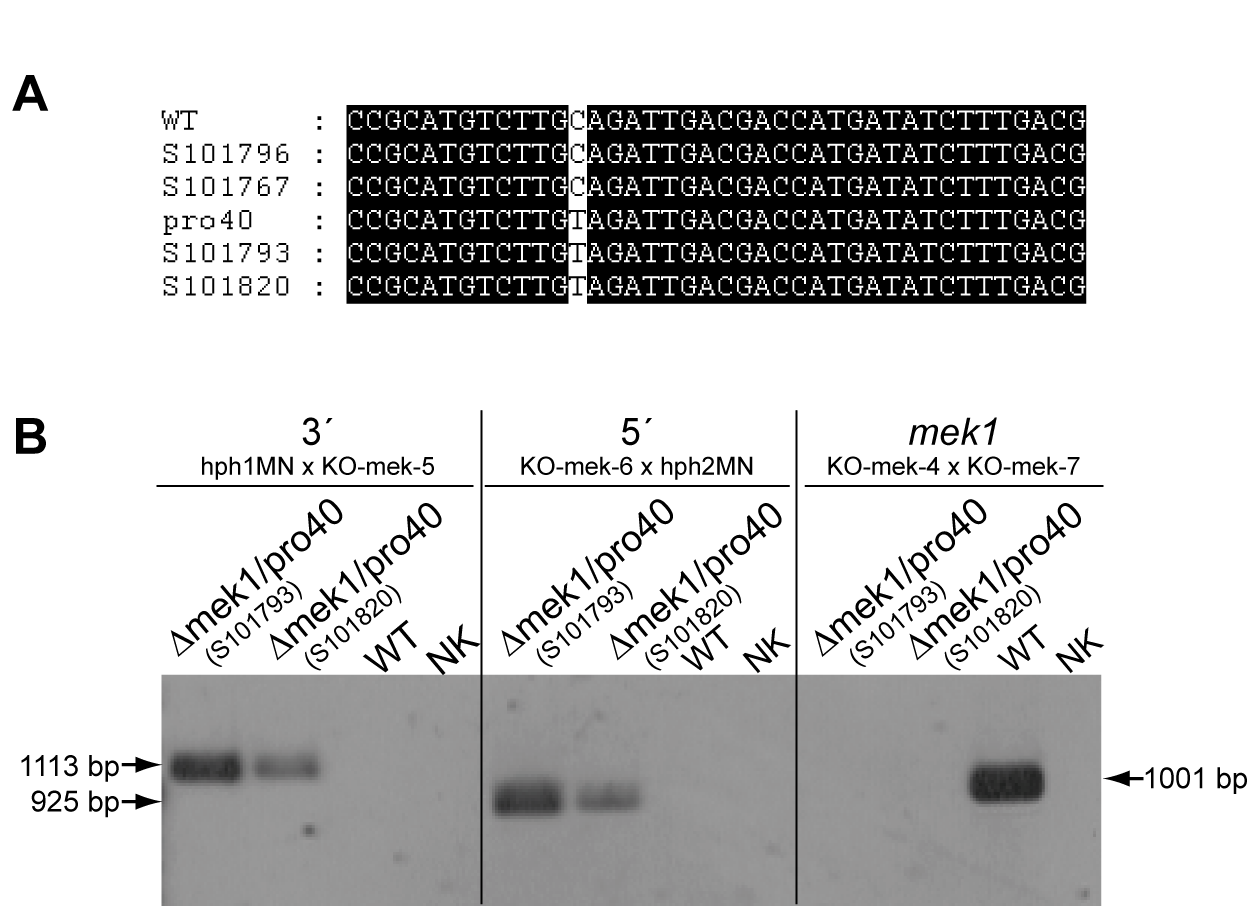

Supplement: Figure S7 — Generation of Δmek1/pro40 double mutants. (A) Sequence analysis of strains from a Δku70/Δmek1 to pro40 cross. Strains S101796 and S101767 show the wildtype (WT) sequence, whereas strains S100793 and S101820 show the pro40 mutation. (B) PCR analysis of Δmek1/pro40 strains S101793 and S101820 to verify the mek1 deletion. WT, wildtype; NK, negative control. Primers are depicted in Figure S3A. (TIF) [file pgen.1004582.s007.tif]

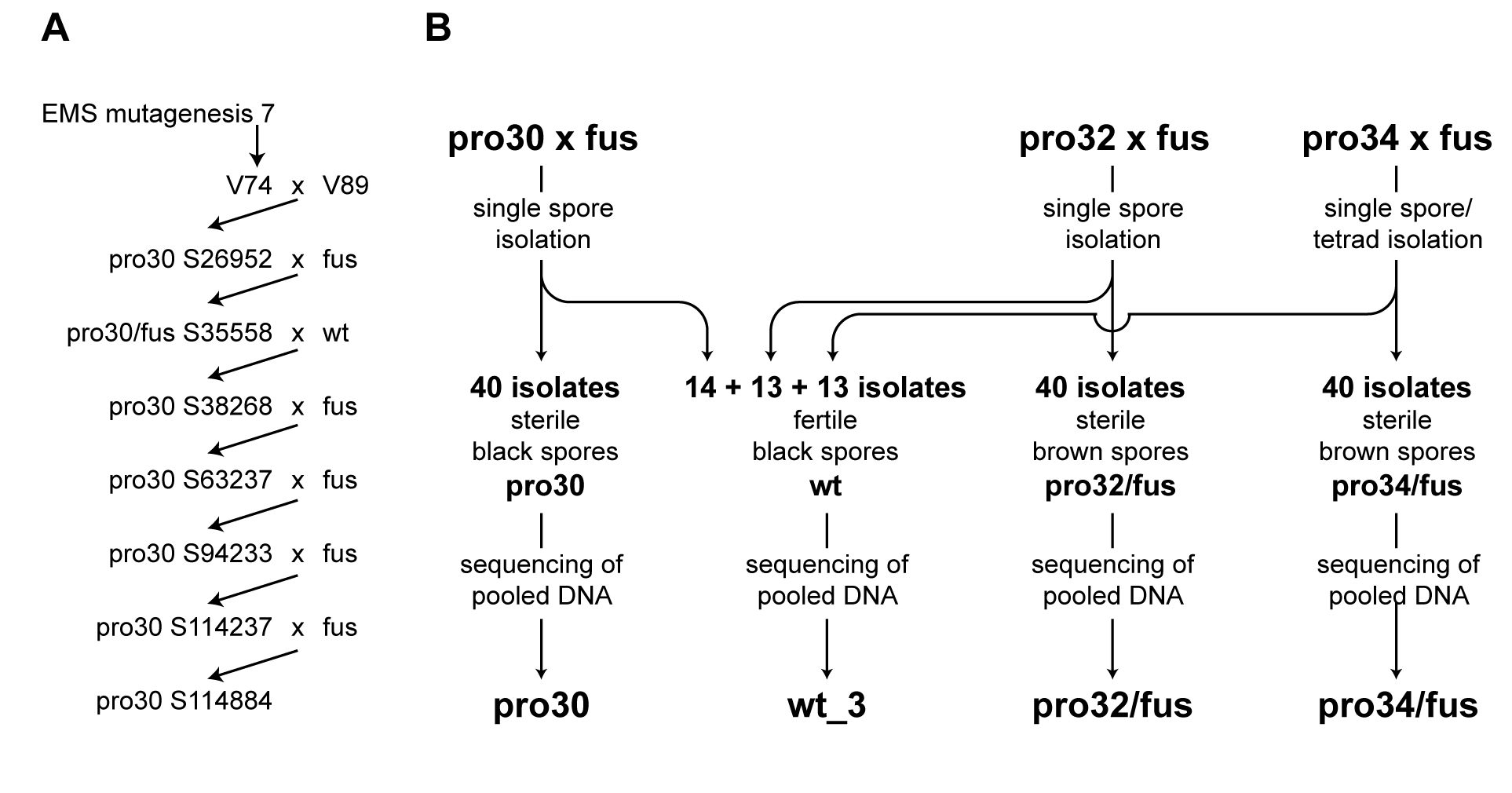

Supplement: Figure S8 — Crossing history of strains for genome sequencing. (A) Crossing history of mutant pro30. Strains were backcrossed to wildtype (wt) or spore color mutant fus [25], which is fertile but produces light-brown instead of black spores. (B) Strategy for whole-genome sequencing of pooled DNA from mutants and wildtype. Mutants pro30, pro32, and pro34 were crossed to spore color mutant fus. Single spore isolates derived from black and light-brown ascospores were screened for fertility and color. For sample pro30, 40 single spore isolates with a sterile phenotype were chosen. For re-sequencing of the wildtype, 40 isolates with a fertile phenotype were chosen from the three crosses. The pooled DNA from 40 single spore isolates for each genotype (pro30 and wt_3) was used for sequencing. (TIF) [file pgen.1004582.s008.tif]

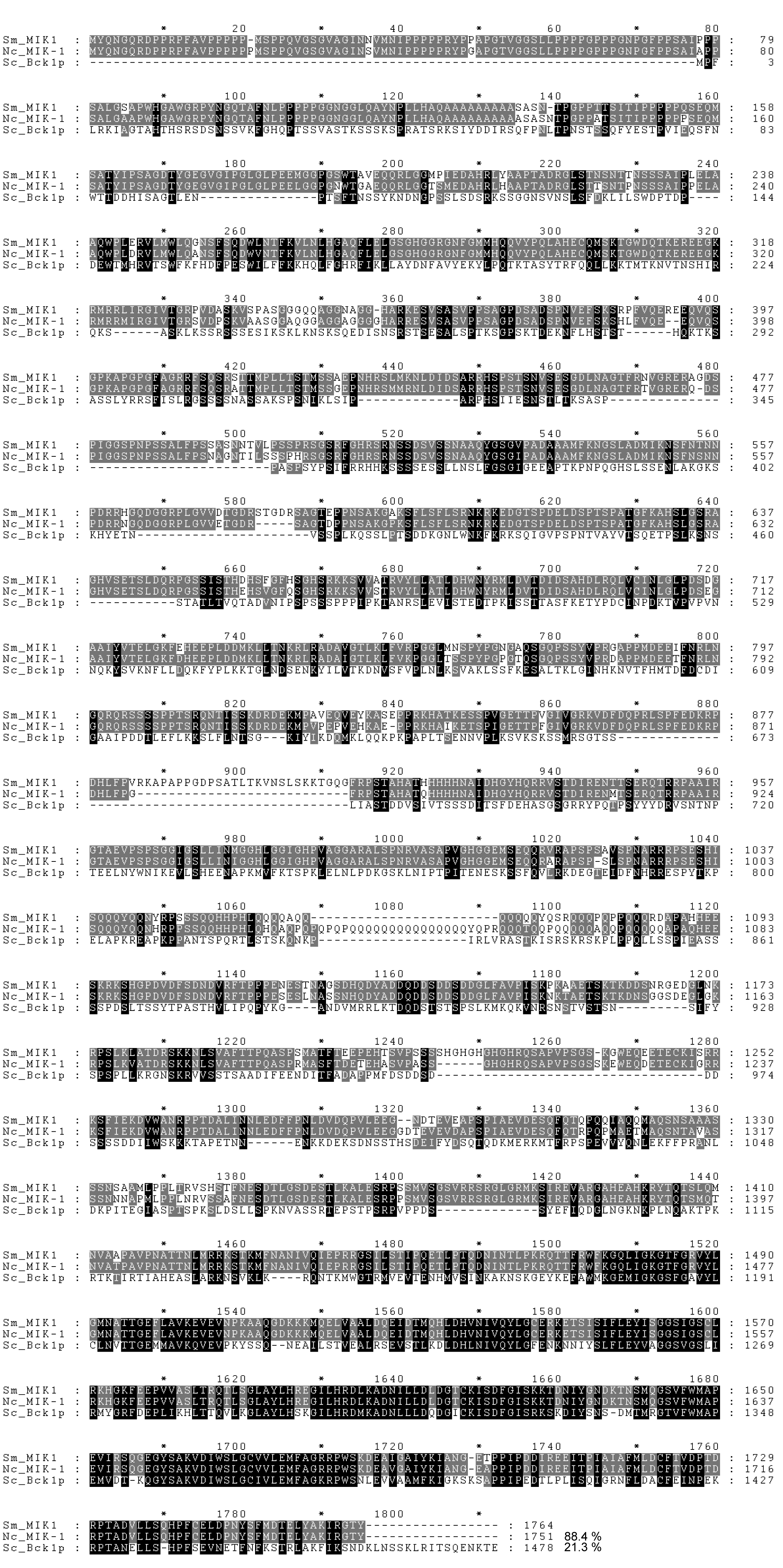

Supplement: Figure S9 — Alignment of MIK1 homologs from S. macrospora, N. crassa, and S. cerevisiae. Alignments were generated with ClustalW (http://www.genome.jp/tools/clustalw/) and edited in GeneDoc. Identity to the S. macrospora proteins is given in percent. Sm_MIK1, CCC09641.1; Nc_MIK-1, XP_959647.2; Sc_Bck1p, EWG95039.1. (TIF) [file pgen.1004582.s009.tif]

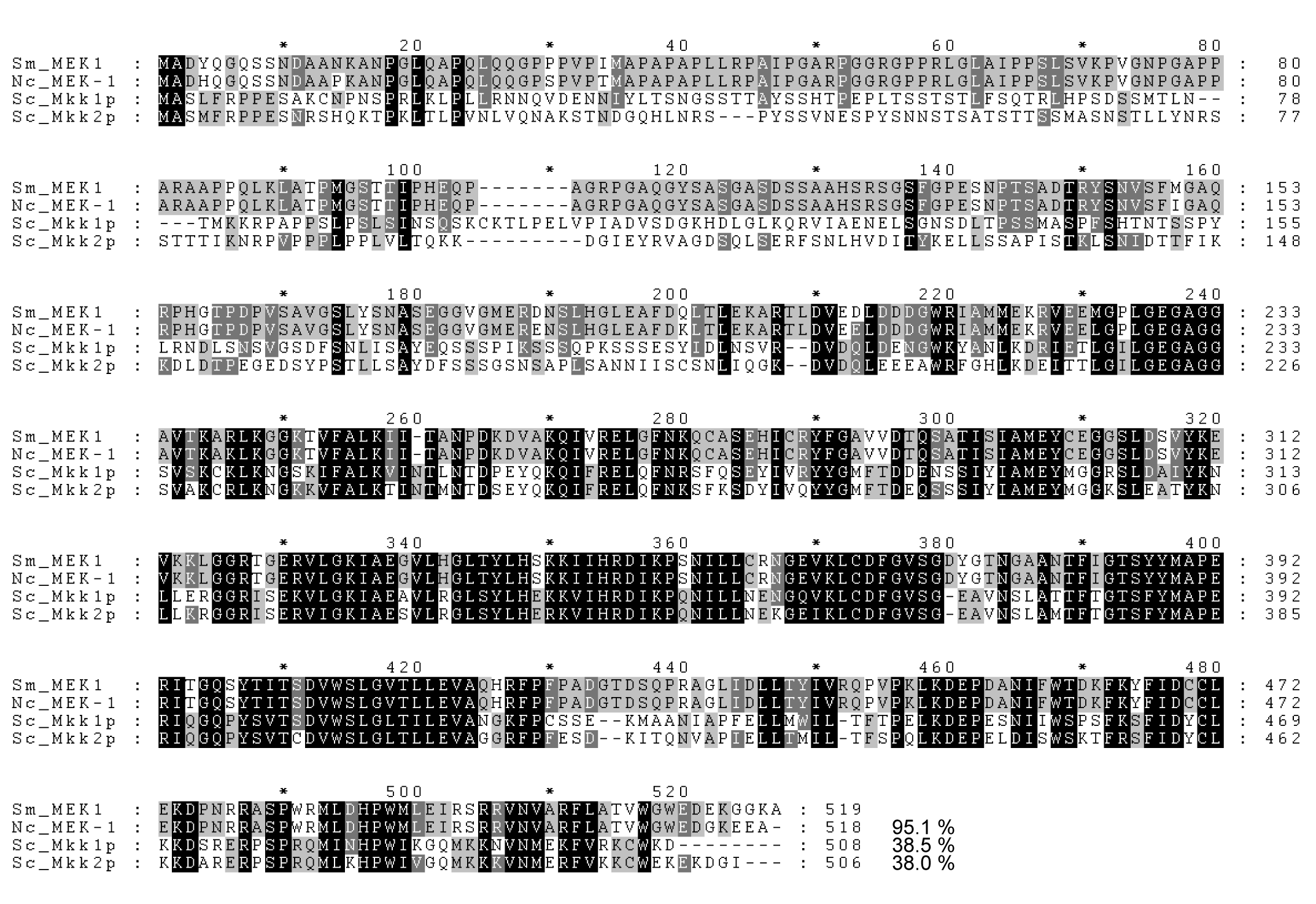

Supplement: Figure S10 — Alignment of MEK1 homologs from S. macrospora, N. crassa, and S. cerevisiae. Alignments were generated with ClustalW (http://www.genome.jp/tools/clustalw/) and edited in GeneDoc. Identity to the S. macrospora proteins is given in percent. Sm_MEK1, CCC11961.1; Nc_MEK-1, XP_957310.2; Sc_Mkk1p, EWG93186.1; Sc_Mkk2p, EWG93010.1. (TIF) [file pgen.1004582.s010.tif]

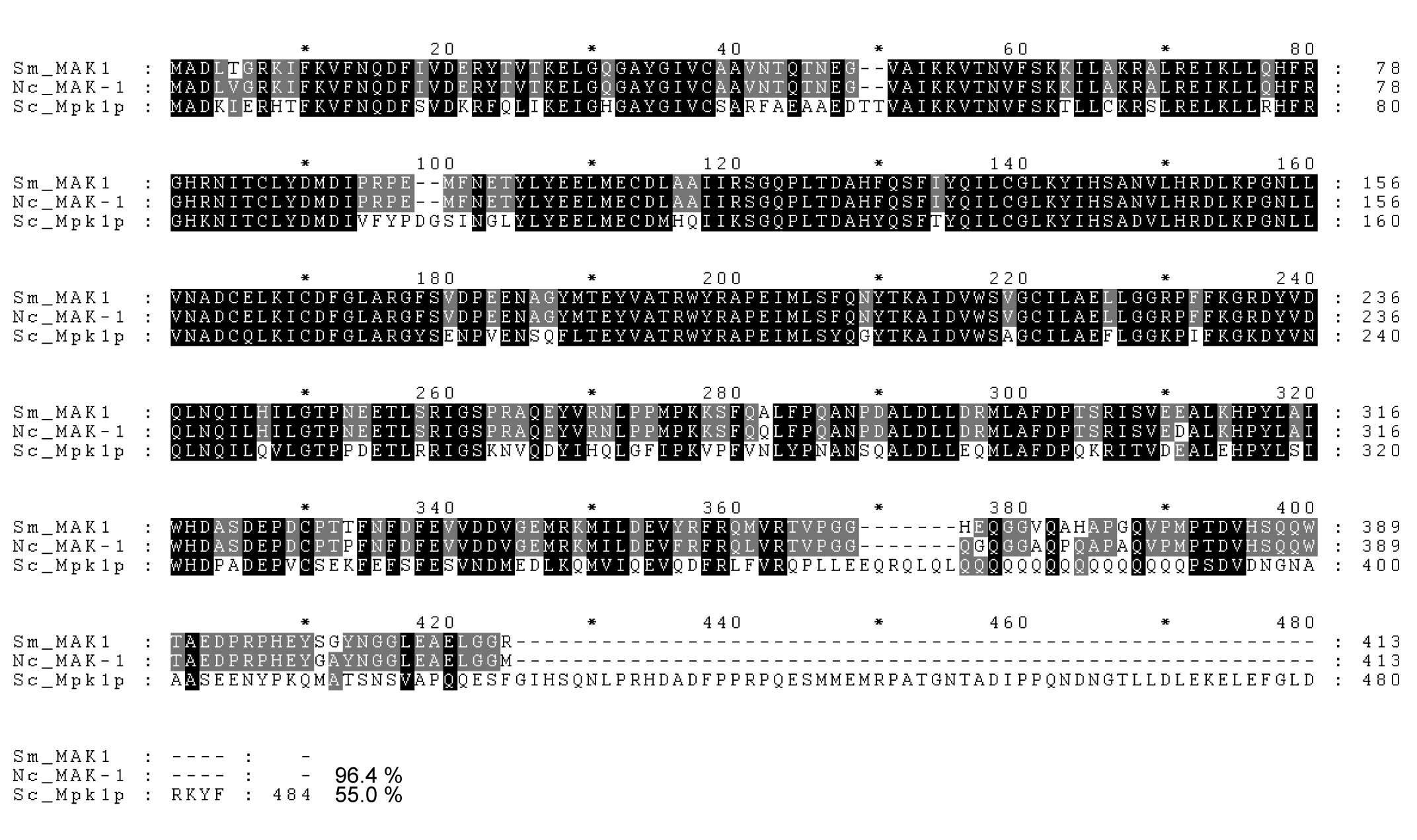

Supplement: Figure S11 — Alignment of MAK1 homologs from S. macrospora, N. crassa, and S. cerevisiae. Alignments were generated with ClustalW (http://www.genome.jp/tools/clustalw/) and edited in GeneDoc. Identity to the S. macrospora proteins is given in percent. Sm_MAK1, CCC12327.1; Nc_MAK-1, EAA28804.2; Sc_Mpk1p, AAB26249.1. (TIF) [file pgen.1004582.s011.tif]
