# Supplementary material for: PRO40 Is a Scaffold Protein of the Cell Wall Integrity Pathway, Linking the MAP Kinase Module to the Upstream Activator Protein Kinase C
Source: PLoS Genet. 2014 Sep 4;10(9):e1004582. doi: 10.1371/journal.pgen.1004582 (PMC4154660; doi:10.1371/journal.pgen.1004582)
Supplement: Table S1 — Summary of sequence reads and small variants from genome sequencing of pro30 and wildtype. (PDF) [file pgen.1004582.s012.pdf]

Table S1. Summary of sequence reads and small variants from genome sequencing of mutant pro30 and wildtype.

Sample	pro30	wt_3
**Genotype**	pro30	wild type
**Total number of reads**	118,469,667	108,513,967
**Total Mb**	6041	5534
**No. of reads mapped to reference genome**	115,716,575	105,047,645
**% of reads mapped to reference genome**	97.7	96.8
**Coverage**	148x	134x
**Number of small variants with coverage > 40 %**	125	146
**Number of these with 100 % penetrance**	2 (one not within coding region, and C to T substitution at position 904 of *SMAC_03673*)	-
