# Supplementary material for: PRO40 Is a Scaffold Protein of the Cell Wall Integrity Pathway, Linking the MAP Kinase Module to the Upstream Activator Protein Kinase C
Source: PLoS Genet. 2014 Sep 4;10(9):e1004582. doi: 10.1371/journal.pgen.1004582 (PMC4154660; doi:10.1371/journal.pgen.1004582)
Supplement: Table S7 — List of putative PRO40 interaction partners identified by yeast two-hybrid screens. (PDF) [file pgen.1004582.s018.pdf]

Table S7. List of putative PRO40 interaction partners identified by yeast two-hybrid screens.

***S. macrospora* locus tag**	**SmI[a]**	**SmII[a]**	**putative function**	***N. crassa***	***S. cerevisiae***
**SMAC_00499**	0	1	RNA recognition motif-containing protein	NCU02950	-
**SMAC_00747**	1	2	SPG20 protein	NCU06989	-
**SMAC_01386**	1	0	60s ribosomal protein l44	NCU00706	RPL42A
**SMAC_01889**	1	0	Unnamed protein product [*Sordaria macrospora*]	NCU00454	-
**SMAC_02183**	0	11	MEK1	NCU06419	MKK1
**SMAC_04037**	1	0	Agmatinase 1	NCU01348	-
**SMAC_05709**	0	1	3 (2) -Bisphosphate nucleotidase	NCU09567	-
**SMAC_07731**	0	1	Unnamed protein product [*Sordaria macrospora*]	NCU07142	-
**SMAC_07925**	2	47	Polyubiquitin	NCU05995	UBI4
**SMAC_08587**	0	2	Unnamed protein product [*Sordaria macrospora*]	-	-
**SMAC_08700**	1	0	Unnamed protein product [*Sordaria macrospora*]	NCU09355	-
**SMAC_09557**	0	4	Het domain protein	NCU10003	-
**SMAC_12623**	1	0	Nicotinate-nucleotide pyrophosphorylase	NCU02998	BNA6

[a] SmI and SmII denote the two *S. macrospora* cDNA libraries generated in this study.
