# Supplementary material for: PRO40 Is a Scaffold Protein of the Cell Wall Integrity Pathway, Linking the MAP Kinase Module to the Upstream Activator Protein Kinase C
Source: PLoS Genet. 2014 Sep 4;10(9):e1004582. doi: 10.1371/journal.pgen.1004582 (PMC4154660; doi:10.1371/journal.pgen.1004582)
Supplement: Table S8 — Strains used in this study. (PDF) [file pgen.1004582.s019.pdf]

Table S8. Strains used in this study.

Strain	Relevant genotype and phenotype[a]	Reference / source
S91327	wild type (wt), fertile	culture collection[b]
S69012	Δ*ku70*::*nat*^{r} fertile	[1]
S84595	*fus1*, fertile, red ascospores	culture collection[b], [2]
S38717	*pro40*, sterile	[3]
S69656	*Δpro40*::*hph*^{r}, sterile	[3]
T184.2NS11	Single spore isolate of S69656 with ectopically integrated pC-FLAG-PRO40; Δ*pro40::hph*^{r}*,* *gpd(p)::pro40::3xFLAG::trpc(t)::hph*^{r}, *nat*^{r}, fertile	this work, [3]
S113578	Single spore isolate; *Δmik1*::*hph*^{r}, sterile	this work
IT1042	Single spore isolate of S1135778 with ectopically integrated pRSnat-gfp-mik1; *Δmik1*::*hph*^{r}*, gpd(p)::gfp::mik1::nat*^{r}, fertile	this work
S101796	Single spore isolate; *Δmek1*::*hph*^{r}, sterile	this work
E292	Single spore isolate of S101796 with ectopically integrated pNTAP-MEK1; *Δmek1*::*hph*^{r}*, gpd(p)::NTAP::mek1::nat*^{r}, fertile	this work
R8501	Single spore isolate; *Δmak1*::*hph*^{r}, sterile	this work
IT1118	Single spore isolate of R8501 with ectopically integrated pNpX-MAK1; *Δmak1*::*hph*^{r}*, xyl(p)::mak1::nat*^{r}, fertile	this work
S101793	*Δmek1*::*hph*^{r}*, pro40*, sterile	this work
E2544	Single spore isolate of S69656 with ectopically integrated pNTAP-MEK1; *Δpro40*::*hph*^{r}*, gpd(p)::NTAP::mek1::nat*^{r}, sterile	this work
R13836	Single spore isolate of S91327 with ectopically integrated pGFP-MIK1_NA; *mik1(p)::gfp::mik1::mik1(t)::nat*^{r}, fertile	this work
R13612	Single spore isolate of S38717 with ectopically integrated pGFP-MIK1_NA; pro40, *mik1(p)::gfp::mik1::mik1(t)::nat*^{r}, sterile	this work
R13747	Single spore isolate of S69656 with ectopically integrated pGFP-MIK1_NA; *Δpro40*::*hph*^{r}*, mik1(p)::gfp::mik1::mik1(t)::nat*^{r}, sterile	this work
R13754	Single spore isolate of S91327 with ectopically integrated pMEK1-GFP_NA; *mek1(p):: mek1::gfp::mek1(t)::nat*^{r}, fertile	this work

Strain	Relevant genotype and phenotype[a]	Reference / source
R13657	Single spore isolate of S38717 with ectopically integrated pMEK1-GFP_NA; *pro40*, *mek1(p):: mek1::gfp::mek1(t)::nat^r*, sterile	this work
R13763	Single spore isolate of S69656 with ectopically integrated pMEK1-GFP_NA; *Δpro40*::*hph^r, mek1(p)::mek1::gfp::mek1(t)::nat^r*, sterile	this work
R13729	Single spore isolate of S91327 with ectopically integrated pMAK1-GFP_NA; *mak1(p):: mak1::gfp::mak1(t)::nat^r*, fertile	this work
R13728	Single spore isolate of S38717 with ectopically integrated pMAK1-GFP_NA; *pro40*, *mak1(p):: mak1::gfp::mak1(t)::nat^r*, sterile	this work
R13793	Single spore isolate of S69656 with ectopically integrated pMAK1-GFP_NA; *Δpro40*::*hph^r, mak1(p):: mak1::gfp::mak1(t)::nat^r*, sterile	this work
S114884	Single spore isolate; *pro30*, sterile	culture collection[b]
T1080C	Primary transformant of S114884 with ectopically integrated pGFP-MIK1_NA; *pro30*, *mik1(p)::gfp::mik1::mik1(t)::nat^r*, fertile	this work
T1094A	Primary transformant of S114884 with ectopically integrated pGFP-MIK1_NA; *pro30*, *mik1(p)::gfp::mik1::mik1(t)::nat^r*, fertile	this work
TES85B-4	Primary transformant of S91327 with ectopically integrated pRH2B; *gpd(p)::h2b::tdTomato::trpC(t)::hph^r*	this work

[a] nat^r, nourseothricin resistant; hph^r, hygromycin resistant.

[b] Department for General and Molecular Botany, Ruhr-Universität, Bochum, Germany.

References

1. Pöggeler S, Kück U (2006) Highly efficient generation of signal transduction knockout mutants using a fungal strain deficient in the mammalian *ku70* ortholog. Gene 378: 1-10.
2. Nowrousian M, Teichert I, Masloff S, Kück U (2012) Whole-genome sequencing of *Sordaria macrospora* mutants identifies developmental genes. G3 (Bethesda) 2: 261-270.
3. Engh I, Wuertz C, Witzel-Schlömp K, Zhang HY, Hoff B, et al. (2007) The WW domain protein PRO40 is required for fungal fertility and associates with Woronin bodies. Eukaryot Cell 6: 831-843.
