# Supplementary material for: PRO40 Is a Scaffold Protein of the Cell Wall Integrity Pathway, Linking the MAP Kinase Module to the Upstream Activator Protein Kinase C
Source: PLoS Genet. 2014 Sep 4;10(9):e1004582. doi: 10.1371/journal.pgen.1004582 (PMC4154660; doi:10.1371/journal.pgen.1004582)
Supplement: Table S9 — Plasmids used in this study. (PDF) [file pgen.1004582.s020.pdf]

Table S9. Plasmids used in this study.

Plasmid	Description	Reference
pA-MAK1	1242 bp full-length *mak1* cDNA in pGADT7, generated by yeast recombination, encodes fusion protein AD-MAK1	this work
pA-MEK1	1560 bp full-length *mek1* cDNA in *Eco*RI and *Bam*HI site in pGADT7, encodes fusion protein AD-MEK1	this work
pA-MEK1_v01	1458 bp *mek1* cDNA fragment corresponding to SMAC_02183 in genome version 01 of *S. macrospora* [1] in *Eco*RI and *Bam*HI site in pGADT7, encodes fusion protein AD-MEK1_v01	this work
pA-MEK1a	324 bp *mek1* cDNA fragment in EcoRI and BamHI site in pGADT7, encodes fusion protein AD-MEK1a (MEK1 1-108)	this work
pA-MEK1b	336 bp *mek1* cDNA fragment in EcoRI and BamHI site in pGADT7, encodes fusion protein AD-MEK1b (MEK1 109-220)	this work
pA-MEK1c	660 bp *mek1* cDNA fragment in EcoRI and BamHI site in pGADT7, encodes fusion protein AD-MEK1c (MEK1 1-220)	this work
pA-MEK1d	900 bp *mek1* cDNA fragment in EcoRI and BamHI site in pGADT7, encodes fusion protein AD-MEK1c (MEK1 221-519)	this work
pA-MIK1	5295 bp *mik1* full-length cDNA in pGADT7, generated by yeast recombination, encodes fusion protein AD-MIK1	this work
pA-PKC1	3441 bp full-length *pkc1*-cDNA in pGADT7, generated by yeast recombination, encodes fusion protein AD-PKC1	this work
pA-PRO40	3961 bp *pro40* full-length cDNA in pGADT7, generated by yeast recombination, encodes fusion protein AD-PRO40	this work
pA-PRO40a	900 bp *pro40* cDNA fragment in *Eco*RI and *Bam*HI site in pGADT7, encodes fusion protein AD-PRO40a (PRO40 1-297)	this work
pA-PRO40AAA	PRO40AAA fragment from pB-PRO40AAA in pA-PRO40AAA, generated by yeast recombination, encodes fusion protein AD-PRO40AAA (PRO40 W575A, W598A, P601A)	this work
pA-PRO40b	861 bp *pro40* cDNA fragment in *Eco*RI and *Bam*HI site in pGADT7, encodes fusion protein AD-PRO40b (PRO40 247-530)	this work
pA-PRO40c	969 bp *pro40* cDNA fragment in *Eco*RI and *Bam*HI site in pGADT7, encodes fusion protein AD-PRO40c (PRO40 481-800)	this work
pA-PRO40d	822 bp *pro40* cDNA fragment in *Eco*RI and *Bam*HI site in pGADT7, encodes fusion protein AD-PRO40d (PRO40 760-1030)	this work
pA-PRO40e	1007 bp pB-PRO40e/EcoRI elution in *Eco*RI site in pGADT7, encodes fusion protein AD-PRO40e (PRO40 989-1316)	this work
pA-RHO1_CA	588 bp *rho1_ca* cDNA in *Eco*RI and *Bam*HI site in pGADT7, encodes AD-RHO1_CA (G15V, C191S)	this work

Plasmid	Description	Reference
pA-RHO1_CI	588 bp *rho1_ci* cDNA in pGADT7, generated by yeast recombination, encodes AD-RHO1_CI (E41I, C191S)	this work
pB-MAK1	1242 bp full-length *mak1* cDNA in *Eco*RI and *Pst*I site in pGBKT7, encodes fusion protein BD-MAK1	this work
pB-MEK1	1560 bp *mek1* cDNA, generated by yeast recombination, encodes fusion protein BD-MEK1	this work
pB-MEK1_v01	1458 bp *mek1* cDNA fragment corresponding to SMAC_02183 in genome version 01 of *S. macrospora* [1] in *Eco*RI and *Bam*HI site of pGBKT7, encodes fusion protein BD-MEK1_v01	this work
pB-MEK1a	324 bp *mek1* cDNA fragment in *Eco*RI and *Bam*HI site of pGBKT7, encodes fusion protein BD-MEK1a (MEK1 1-108)	this work
pB-MEK1b	336 bp *mek1* cDNA fragment in *Eco*RI and *Bam*HI site of pGBKT7, encodes fusion protein BD-MEK1b (MEK1 109-220)	this work
pB-MEK1c	660 bp *mek1* cDNA fragment in *Eco*RI and *Bam*HI site of pGBKT7, encodes fusion protein BD-MEK1c (MEK1 1-220)	this work
pB-MEK1d	900 bp *mek1* cDNA fragment in *Eco*RI and *Bam*HI site of pGBKT7, encodes fusion protein BD-MEK1c (MEK1 221-519)	this work
pB-MIK1	5295 bp *mik1* full-length cDNA in pGBKT7, generated by yeast recombination, encodes fusion protein BD-MIK1	this work
pB-PKC1	3441 bp *pkc1* full-length cDNA in pGBKT7, generated by yeast recombination, encodes fusion protein BD-PKC1	this work
pB-PRO40	3961 bp *pro40* full-length cDNA in *Eco*RI and *Pst*I site in pGBKT7, encodes fusion protein BD-PRO40	this work
pB-PRO40a	900 bp *pro40* cDNA fragment in *Eco*RI and *Bam*HI site in pGBKT7, encodes fusion protein BD-PRO40a (PRO40 1-297)	this work
pB-PRO40AAA	yeast recombination of a mutated *pro40* cDNA fragment in *Sca*I site of pB-PRO40, encodes fusion protein BD-PRO40AAA (PRO40 W575A, W598A, P601A)	this work
pB-PRO40b	861 bp *pro40* cDNA fragment in *Eco*RI and *Bam*HI site in pGBKT7, encodes fusion protein BD-PRO40b (PRO40 247-530)	this work
pB-PRO40c	969 bp *pro40* cDNA fragment in *Eco*RI and *Bam*HI site in pGBKT7, encodes fusion protein BD-PRO40c (PRO40 481-800)	this work
pB-PRO40d	822 bp *pro40* cDNA fragment in *Eco*RI and *Bam*HI site in pGBKT7, encodes fusion protein BD-PRO40d (PRO40 760-1030)	this work
pB-PRO40e	1007 bp *pro40* cDNA fragment in *Eco*RI site in pGBKT7, encodes fusion protein BD-PRO40e (PRO40 989-1316)	this work
pB-RHO1_CA	588 bp *rho1_ca* cDNA in *Eco*RI and *Bam*HI site in pGBKT7, encodes BD-RHO1_CA (G15V, C191S)	this work

Plasmid	Description	Reference
pB-RHO1_CI	588 bp *rho1_ci* cDNA in pGBKT7, generated by yeast recombination, encodes BD-RHO1_CI (E41I, C191S)	this work
pC-FLAG-PRO40	*gpd(p)::pro40::3xFLAG::trpC(t), trpC(p)::hyg*	[2]
pDrive	PCR cloning vector	QIAGEN, Hilden, Germany
pDrivehph	hygromycin resistance cassette in pDrive	[3]
pDrive-Hyg	in pDrive	Godehardt and Kück, unpublished
pDS21	*gpd(p)::NTAP::trpC(t)*, nat	[4]
pDS23	*ura3(p)::URA, gpd(p)::egfp::trpC(t), trpC(p)::nat*	M. Nowrousian, unpublished
pGADT7	*ADH1(p)::gal4-AD::LEU2*	Clontech
pGBKT7	*ADH1(p)::gal4-BD::TRP1*	Clontech
pGFP-MIK1_NA	*mik1(p)::egfp::mik1::mik1(t)::nat*	this work
pKO-MAK1	1000 bp 5' and 3' region of *mak1* flanking an *hph* resistance cassette in pRS426	this work
pKO-MEK1	832 bp 5' and 913 bp 3' region of *mek1* in *Sna*BI / *Bam*HI and *Xba*I / *Apa*I sites of pDrive-Hyg	this work
pKO-MIK1	1000 bp 5' and 3' region of *mik1* flanking an *hph* resistance cassette in pRS426	this work
pMAK1-GFP_NA	*mak1(p)::mak1::egfp::mak1(t)::nat*	this work
pMEK1-GFP_NA	*mek1(p)::mek1::egfp::mek1(t)::nat*	this work
pNpX-GFP	*Smxyl(p)::egfp::trpC(t)::nat*	[5]
pNpX-MAK1	*Smxyl(p)::mak1::trpC(t)::nat*	this work
pNTAP-MEK1	*gpd(p)::NTAP::mek1::trpC(t), ura3(p)::URA, trpC(p)::nat*	this work
pNTAP-mik1	*gpd(p)::TAP::mik1::trpC(t)::nat*	this work
pRH2B	*gpd(p)::h2b::tdTomato::trpC(t)::hph^r*	Teichert and Kück, unpublished
pRSnat	derivative of plasmid pRS426 with nourseothricin resistance gene *nat1* controlled by the *Aspergillus nidulans trpC* promoter	[6]
pRSnat-gfp-mik1	*gpd(p)::egfp::mik1::trpC(t)::nat*	this work
pRSnat-MAK1-GFP	*mak1(p)::mak1::egfp::mak1(t)::nat*	this work
pRSnat-MEK1-GFP_V3	*gpd(p)::mek1::egfp::trpC(t)::nat*	this work

References

1. Nowrousian M, Stajich JE, Chu M, Engh I, Espagne E, et al. (2010) *De novo* assembly of a 40 Mb eukaryotic genome from short sequence reads: *Sordaria macrospora*, a model organism for fungal morphogenesis. PLoS Genet 6: e1000891.
2. Engh I, Würtz C, Witzel-Schlömp K, Zhang HY, Hoff B, et al. (2007) The WW domain protein PRO40 is required for fungal fertility and associates with Woronin bodies. Eukaryot Cell 6: 831-843.
3. Nowrousian M, Cebula P (2005) The gene for a lectin-like protein is transcriptionally activated during sexual development, but is not essential for fruiting body formation in the filamentous fungus *Sordaria macrospora*. BMC Microbiol 5: 64.
4. Gesing S, Schindler D, Fränzel B, Wolters D, Nowrousian M (2012) The histone chaperone ASF1 is essential for sexual development in the filamentous fungus *Sordaria macrospora*. Mol Microbiol 84: 748-765.
5. Bloemendal S, Löper D, Terfehr D, Kopke K, Kluge J, et al. (2014) Tools for advanced and targeted genetic manipulation of the beta-lactam antibiotic producer *Acremonium chrysogenum*. J Biotechnol 169: 51-62.
6. Klix V, Nowrousian M, Ringelberg C, Loros JJ, Dunlap JC, et al. (2010) Functional characterization of MAT1-1-specific mating-type genes in the homothallic ascomycete *Sordaria macrospora* provides new insights into essential and nonessential sexual regulators. Eukaryot Cell 9: 894-905.
