# Supplementary material for: PRO40 Is a Scaffold Protein of the Cell Wall Integrity Pathway, Linking the MAP Kinase Module to the Upstream Activator Protein Kinase C
Source: PLoS Genet. 2014 Sep 4;10(9):e1004582. doi: 10.1371/journal.pgen.1004582 (PMC4154660; doi:10.1371/journal.pgen.1004582)
Supplement: Table S10 — Oligonucleotides used in this study. (PDF) [file pgen.1004582.s021.pdf]

**Table S10.** Oligonucleotides used in this study.

| **Oligonucleotide** | **Sequence (5'- 3')[a]** |
|---|---|
| 2183-3fw-gfp | TGTACAAGTAAAGCGGCCGCTCTAGATGTGTCTGCTGTGGTGGAAATAATCG |
| 2183-3rv_IT | GGATAACAATTTCACACAGGAAACAGCCATATCACTGCACTCCAGTCG |
| 2183-5fw_IT | ACGCCAGGGTTTTCCCAGTCACGACGCGCCGTTCTATCGTTGAGTTGC |
| 3673-10-AD | GATTCATCTGCAGCTCGAGCTCGATTTAATAAGTCCCCCTAATCTTAGCATACAGC |
| 3673-10-BD | AGTTATGCGGCCGCTGCAGGTCGACTTAATAAGTCCCCCTAATCTTAGCATACAGC |
| 3673-11 | CGTTCTTCTCTCGCAACACC |
| 3673-1-AD | CGCTCATATGGCCATGGAGGCCAGTATGTACCAAAATGGCCAGAGGGACCC |
| 3673-1-BD | GGACCTGCATATGGCCATGGAGGCCATGTACCAAAATGGCCAGAGGGACCC |
| 3673-2 | CGGGCGCTGTCTATATCCAGGTTC |
| 3673-3 | GCAGGAGATTCTCCCAATCGCGC |
| 3673-3fw | GCCCAAAAATGCTCCTTCAATATCAGTTGCTAATTCACACGCTATCGCCATCATC |
| 3673-3rv | GCGGATAACAATTTCACACAGGAAACAGC**GAATTC**GTTGAGGAAAAGTCCCAAGCCGCAC |
| 3673-4 | GCTAGGTCGGAATCCCTGTCCAG |
| 3673-5 | CCGACTCTCGCCGTTCGAGGAC |
| 3673-5fw | GTAACGCCAGGGTTTTCCCAGTCACGACG**GAATTC**ACTTTGGTACTACTGAGTGTCGTGA |
| 3673-5rv | CGAGGGCAAAGGAATAGGGTTCCGTTGAGGGGCCGGAGGCAACACCTAGGTACGG |
| 3673-5rv-gfp | ACAGCTCCTCGCCCTTGCTCACCATGGCCGGAGGCAACACCTAGGTACGG |
| 3673-6 | GAACCGCAAAGAGGCCATCGTCC |
| 3673-7 | GCGATAACGATGTGCGCTTCACCC |
| 3673-8 | GCAGCGACGTTTGCGTGTAGCGC |
| 3673-9 | CGAGTCTACGCTCAAGGCGCTGG |
| 3673KOvp1IT | TGTCGTCGGGTCATGATAGG |
| 3673KOvp2IT | AGCCCCATGACACTAAAACC |
| 3673KOvp3IT | CGACTCCAGAACCGACTTGC |
| 40-6 | ACCGCCGCCTTTGCCTGAGGGTgccATTGCCCATCTTGAC |
| 40-7 | GGAAATGGGGTTGGGACCCTTggcGAACTCtgcTTGTGTGGCCTGGGTG |
| 4666-01-AD | ATATGGCCATGGAGGCCAGT**GAATTC**ATGAACGACGAAGACAAGG |
| 4666-01-BD | TGCATATGGCCATGGAGGCC**GAATTC**ATGAACGACGAAGACAAGG |
| 4666-02 | TCTGGCCATAAGGCTCCAG |
| 4666-03 | CGCCATCCCAGGTTGTTTC |
| 4666-04 | GTACATTGGTGAGACCTGC |
| 4666-05 | GCGGACTACGCTAATATCG |
| 4666-06-AD | TGCAGCTCGAGCTCGAT**GGATCC**CGTTTACTCAAAGTCGGCAGTG |
| 4666-06-BD | ATGCGGCCGCTGCAGGTCGAC**GGATCC**TTACTCAAAGTCGGCAGTG |
| 5504-3fw-gfp | AGTAAAGCGGCCGCTCTAGAACTAGTGCGATGAGCGAAGGAAACAAAAGG |

| Oligonucleotide | Sequence (5'- 3')[a] |
|---|---|
| 5504-3fwIT | GCCCAAAAATGCTCCTTCAATATCAGTTGCGCGATGAGCGAAGGAAACAAAAGG |
| 5504-3rv-IT | GCGGATAACAATTTCACACAGGAAACAGC**CTCGAG**GAGGTCCAGCAGGCCTCGGACAAGA |
| 5504-5fw | GTAACGCCAGGGTTTTCCCAGTCACGACG**CTCGAG**GGACTAGTATCGTCGTGTTGTTGAT |
| 5504-5rv | CGAGGGCAAAGGAATAGGGTTCCGTTGAGGGGCGAATGTGTGTATGTAGTGTTTC |
| 5504KOvp1neu | AGTGACATTTGGCAGGCAGG |
| 5504KOvp2IT | CGAATGGAATGGTGGAGAGC |
| 5504KOvp3 | GCTCGTCGACAATGAAGTCC |
| 5504-NcoI-fw | **CCATGGC**TGATCTCACGGGTCG |
| 5504-NcoI-rv2 | **CCATGG**TCCTACCACCAAGCTCGGC |
| 6419-10 | **GGATCC**CTATGCCTTCCCTCCCTTC |
| 6419-7 | CTAGTGTCCTCGCTTTCTCC |
| 6419-9 | **GAATTC**ATGGCACCAGCCCCCGC |
| AD-40-for1 | TATGGCCATGGAGGCCAGTGAATTCATGTCTCGACCCCGCGGTTTTC |
| AD-40-for2 | AGGACGTTGCAGGGGATTGC |
| AD-40-rev1 | TAGAGGCTTGGGAACAGCTC |
| AD-40-rev2 | TTTTCAGTATCTACGATTCATATCCCTAATGCCCATACTCCAAATGC |
| CTAP-mak1-fw | |
| GFP-mak1-rv | AACAGCTCCTCGCCCTTGCTCACCATCCTACCACCAAGCTCGG |
| gfp-mek-rev | AACAGCTCCTCGCCCTTGCTCACCATTGCCTTCCCTCCCTTCTCATC |
| hph-1 | CTGTCGAGAAGTTTCTGATCG |
| hph1MN | CGATGGCTGTGTAGAAGTACTCGC |
| hph2MN | ATCCGCCTGGACGACTAAACCAA |
| hph2neu2010 | GCCTCCAGAAGAAGATGTTG |
| hphfor | CGTTAACTGGTTCCCGGTCG |
| HR-mak1-for | ATATGGCCATGGAGGCCAGTGAATTCATGGCTGATCTCACGGGTCGCAAGATC |
| HR-mak1-rev | GCGGGGTTTTTCAGTATCTACGATTCATCTGCAGCTAACCAAACATTGAGATCAACCCCAG |
| KO-mek-1 | **GGATCC**TATGGGAACGGGGGGTGGTC |
| KO-mek-2 | **TACGTA**ACCTACGAGCCTACCTACCG |
| KO-mek-3 | **TCTAGA**TGTGTCTGCTGTGGTGG |
| KO-mek-4 | **GGGCCC**GCAACCTTTCTCTTAAGTG |
| KO-mek-5 | CGGCATCTGTACACTCCAC |
| KO-mek-6 | CACGTCTACACCATATCACTTCG |
| KO-mek-7 | GGAAGATGAGAAGGGAGGG |
| mek1_F1-fw | **GAATTC***ATG*GCCGACTACCAAGGTC |
| mek1_F1-rv | **GGATCC***CTA*TGGTCGTCCGGCAGGTTG |
| mek1_F2-fw | **GAATTC***ATG*GGTGCCCAAGGCTACAGC |

| Oligonucleotide | Sequence (5’- 3’)[a] |
|---|---|
| mek1_F2-rv | **GGATCC***CTA*TCTCTTCTCCATCATGGC |
| mek1_F4-fw | **GAATTC***ATG*GTCGAGGAGATGGGACCC |
| mek-BamHI-fw | **GGATCC**ATGGCGCCAGCCCCCGCACC |
| mik1_egfp_rev | AGGGTCCCTCTGGCCATTTTGGTACATCTTGTACAGCTCGTCCATGC |
| NTAP-mak-rv | GATTTCAGTAACGTTAAGTGGATCATTACCTACCACCAAGCTCGGCC |
| NTAP-mek-BamHI-rv | **GGATCC**CGCTATGCCTTCCCTCCCTT |
| NTAP-mik-fw | CGTCGACTCCATGGGGTACACTAGTATGTACCAAAATGGCCAGAG |
| NTAP-mik-rv | GATTTCAGTAACGTTAAGTGGATCATTAATAAGTCCCCCTAATCTTAGC |
| pAD-2 | ATTGAGATGGTGCACGATGC |
| pADfor96er | GATGATGAAGATACCCCAC |
| pAD-FPneu | TGGTTGGACGGACCAAAC |
| Pgpd_egfp_for | CGCAGCTTGACTAACAGCTACAGATCTAAGCTTATGGTGAGCAAGGGCGAGG |
| Pgpd-mek1_V3 | AGCTACCCCGCTTGAGCAGACATCACC**ATG**GCCGACTACCAAGGTCAAAGCAGC |
| Pxyl-mak-for | TCTAGGCCTAACCAGAAACCGCCAACATGGCTGATCTCACGGGTCG |
| rho1_CA-for | **GAATTC**ATGTCTGCTGAACTCCGCCGAAAGCTCGTCATCGTTGGCGATGtTGCCTGC |
| rho1_CA-rev | **GGATCC**TTAGACCGAGCTCTTGgAGAGGC |
| rho1_CI-for | ACGCTCATATGGCCATGGAGGCCAGT**GAATTC**ATGTCTGCTGAACTCCGCCG |
| rho1_CIint-for | CatcAACTACGTCGCCGATG |
| rho1_CIint-rev | ACCTCAACATCGGCGACGTAGTTgatGAAAACGGTAGGGACATAGACC |
| rho1_CI-rev | CGATTCATCTGCAGCTCGAGCTCGAT**GGATCC**TTAGACCGAGCTCTTGgAGAGGC |
| Y2H-01 | A**GAATTC**ACTCCGAACCCAACCTCCCG |
| Y2H-03 | A**GAATTC**CGCACCCAGACAAGAGTAATGGCCG |
| Y2H-04 | AGACGTCCTAATGCCCATACTCCAAATGC |
| Y2H-05 | A**GAATTC**ATGTCTCGACCCCGCGGTTT |
| Y2H-06neu | A**CTGCAG**CTAATGCCCATACTCCAAATGCGGCAC |
| Y2H-07 | **GGATCC***TCA*GAGACTCGTGAGGGTTGC |
| Y2H-08 | **GAATTC**TCAACGGCTTCGACTTCGACC |
| Y2H-09 | **GGATCC***TCA*TCCGGGGTGTTGTTGAGC |
| Y2H-10 | **GAATTC**GAGCGCTGGACCTACGCC |
| Y2H-11 | **GGATCC***TCA*CAACCTCGGGTAGACCAC |
| Y2H-12 | **GGATCC***TCA*GCTGGGAGCAAAATCATTCC |
| Y2H-13 | **GAATTC**ATGTCTCGACCCCGCGG |

[a] Restriction enzyme sites are denoted in bold letters, italics indicate additional start or stop codons, lowercase letters indicate mutations, and sequences showing homology to target vectors for yeast recombination are underlined.
