# Supplementary material for: PRO40 Is a Scaffold Protein of the Cell Wall Integrity Pathway, Linking the MAP Kinase Module to the Upstream Activator Protein Kinase C
Source: PLoS Genet. 2014 Sep 4;10(9):e1004582. doi: 10.1371/journal.pgen.1004582 (PMC4154660; doi:10.1371/journal.pgen.1004582)
Supplement: Text S1 — Supplementary methods. (PDF) [file pgen.1004582.s022.pdf]

Text S1. Supplementary Methods.

Mapping

Reads were mapped onto the *S. macrospora* reference genome (version 02; Teichert *et al.*, 2012) with BWA (Li and Durbin, 2009). Reads were cleaned beforehand to remove reads with undetermined bases.

Generate index for the *S. macrospora* genome with

```
./bwa index Sm_v02_genome.fasta
```

Map each of the two files (singlets and pairs) for each genome

- for singlets:

```
./bwa aln Sm_v02_genome.fasta
pro30_trim_fastq_cleaned_singlets.fastq >
pro30_trim_fastq_cleaned_singlets.sai

./bwa samse Sm_v02_genome.fasta
pro30_trim_fastq_cleaned_singlets.sai
pro30_trim_fastq_cleaned_singlets.fastq >
pro30_trim_fastq_cleaned_singlets.sam
```

- for pairs:

```
./bwa aln Sm_v02_genome.fasta
pro30_trim_fastq_cleaned_pairs.fastq_read1.fastq >
pro30_trim_fastq_cleaned_pairs.fastq_read1.sai

./bwa aln Sm_v02_genome.fasta
pro30_trim_fastq_cleaned_pairs.fastq_read2.fastq >
pro30_trim_fastq_cleaned_pairs.fastq_read2.sai

./bwa sampe Sm_v02_genome.fasta
pro30_trim_fastq_cleaned_pairs.fastq_read1.sai
pro30_trim_fastq_cleaned_pairs.fastq_read2.sai
pro30_trim_fastq_cleaned_pairs.fastq_read1.fastq
pro30_trim_fastq_cleaned_pairs.fastq_read2.fastq >
pro30_trim_fastq_cleaned_pairs.sam
```

Downstream processing

Extraction of small sequence variants was performed with SAMtools (Li *et al.*, 2009).

Create index Sm_v02_genome.fasta.fai for reference sequence with:

```
./samtools faidx Sm_v02_genome.fasta
```

Import SAM file from BWA and convert to BAM file with:

```
./samtools import Sm_v02_genome.fasta.fai
pro30_trim_fastq_cleaned_pairs.sam
pro30_trim_fastq_cleaned_pairs.bam

./samtools import Sm_v02_genome.fasta.fai
pro30_trim_fastq_cleaned_singlets.sam
pro30_trim_fastq_cleaned_singlets.bam
```

Sort BAM file (necessary for fast access, sorted by reference sequence contigs etc.) with:

```
./samtools sort pro30_trim_fastq_cleaned_pairs.bam
  pro30_trim_fastq_cleaned_pairs_sorted

./samtools sort pro30_trim_fastq_cleaned_singlets.bam
  pro30_trim_fastq_cleaned_singlets_sorted
```

Merge BAM files:

```
./samtools merge pro30_all_sorted.bam
pro30_trim_fastq_cleaned_singlets_sorted.bam
pro30_trim_fastq_cleaned_pairs_sorted.bam
```

For variant callling (SNPs, indels) use SAMtools mpileup and bcftools (-C option for consensus sequence, -u output is bam file / -b output is BCF format, -v only variants are reported, not complete consensus for all bases, even those that are the same as the reference, -c variant calling options, -g call per-sample genotypes at variant sites):

```
./samtools mpileup -C 50 -u -f Sm_v02_genome.fasta
pro30_all_sorted.bam | bcftools view -bvcg - >
pro30_pileup.bcf
```

Filter raw variant calls with vcfutils varFilter. Set -D option (maximum read depth) according to the average read depth (ca. 2x coverage; here 300).

```
bcftools view pro30_pileup.bcf | vcfutils.pl varFilter -D 300
> pro30_pileup_filter.vcf
```

Acquire final variants by setting a quality threshold with awk. Here, the quality threshold for indels is 25 and 10 for substitutions.

```
awk '($8~/^INDEL/&&$6>=25)||($8!~/^INDEL/&&$6>=10)'
pro30_pileup_filter.vcf > pro30_pileup_filter_threshold.txt
```
